# Birthweight, BMI in adulthood and latent autoimmune diabetes in adults: a Mendelian randomisation study

**DOI:** 10.1007/s00125-022-05725-2

**Published:** 2022-05-23

**Authors:** Yuxia Wei, Yiqiang Zhan, Josefin E. Löfvenborg, Tiinamaija Tuomi, Sofia Carlsson

**Affiliations:** 1 Institute of Environmental Medicine, Karolinska Institutet, Stockholm, Sweden; 2 School of Public Health (Shenzhen), Sun Yat-Sen University, Shenzhen, China; 3 Department of Endocrinology, Helsinki University Hospital, Helsinki, Finland; 4 Institute for Molecular Medicine Finland FIMM and Research Programs Unit, Diabetes and Obesity, University of Helsinki, Helsinki, Finland; 5 Folkhalsan Research Center, Helsinki, Finland; 6 Lund University, Malmö, Sweden

**Keywords:** Epidemiology, Genetics, Human, Weight regulation and obesity

## Abstract

**Aims/hypothesis:**

Observational studies have found an increased risk of latent autoimmune diabetes in adults (LADA) associated with low birthweight and adult overweight/obese status. We aimed to investigate whether these associations are causal, using a two-sample Mendelian randomisation (MR) design. In addition, we compared results for LADA and type 2 diabetes.

**Methods:**

We identified 43 SNPs acting through the fetal genome as instrumental variables (IVs) for own birthweight from a genome-wide association study (GWAS) of the Early Growth Genetics Consortium (EGG) and the UK Biobank. We identified 820 SNPs as IVs for adult BMI from a GWAS of the UK Biobank and the Genetic Investigation of ANthropometric Traits consortium (GIANT). Summary statistics for the associations between IVs and LADA were extracted from the only GWAS involving 2634 cases and 5947 population controls. We used the inverse-variance weighted (IVW) estimator as our primary analysis, supplemented by a series of sensitivity analyses.

**Results:**

Genetically determined own birthweight was inversely associated with LADA (OR per SD [~500 g] decrease in birthweight 1.68 [95% CI 1.01, 2.82]). In contrast, genetically predicted BMI in adulthood was positively associated with LADA (OR per SD [~4.8 kg/m^2^] increase in BMI 1.40 [95% CI 1.14, 1.71]). Robust results were obtained in a range of sensitivity analyses using other MR estimators or excluding some IVs. With respect to type 2 diabetes, the association with birthweight was not stronger than in LADA while the association with adult BMI was stronger than in LADA.

**Conclusions/ interpretation:**

This study provides genetic support for a causal link between low birthweight, adult overweight/obese status and LADA.

**Graphical abstract:**

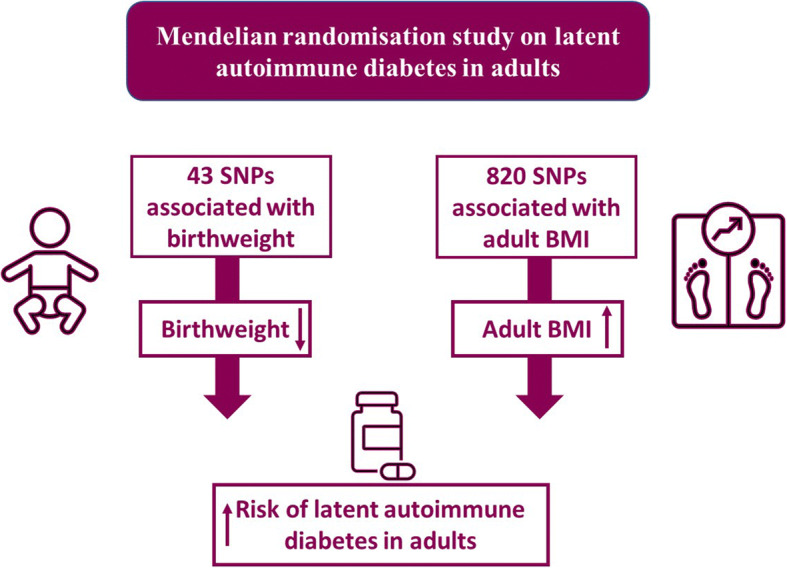

**Supplementary Information:**

The online version of this article (10.1007/s00125-022-05725-2) contains peer-reviewed but unedited supplementary material.

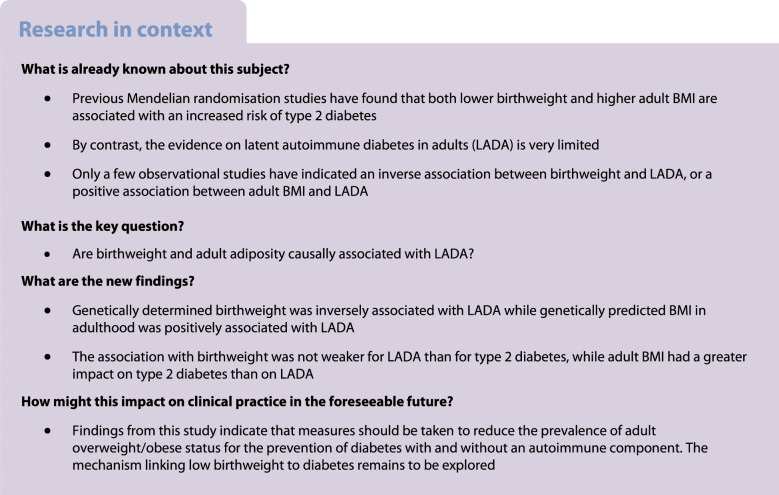



## Introduction

Latent autoimmune diabetes in adults (LADA) is a hybrid form of diabetes. Genetically, it is closely related to type 1 diabetes with a strong link to HLA genotype [1, 2], while many of its clinical features such as the metabolic syndrome are shared with type 2 diabetes [2]. LADA is characterised by pancreatic autoantibodies and its slow progression to insulin dependence, and is usually restricted to adults [2] although a similar phenomenon has been described in younger individuals (latent autoimmune diabetes in the young [LADY]) [3]. Autoantibody testing is required to separate LADA from type 2 diabetes. Around 5–14% of the adults who have been diagnosed with type 2 diabetes in Europe, North America and Asia have pancreatic autoantibodies [2].

Risk factors for type 2 diabetes have been studied extensively [4, 5] and the disease can be prevented or postponed by maintaining a healthy weight and physical activity [6]. In comparison, the evidence on environmental/lifestyle risk factors for LADA is limited [7]. However, we have previously reported an increased risk of LADA in relation to overweight/obese status [8] and low birthweight [9], in line with findings in type 2 diabetes [10, 11]. Observational studies are prone to residual confounding and reverse causation, while randomised control trials may be unfeasible for studying some risk factors of diseases. Individuals’ genotypes are randomly assigned from their parents before conception and thus are less likely to suffer from confounding or reverse causation [12]. Taking advantage of the natural experiments, Mendelian randomisation (MR) studies use genetic variants as instrumental variables (IVs) for an environmentally modifiable exposure to make causal inference about the outcome [12].

Our aim was to investigate whether low birthweight and adult adiposity are implicated in the aetiology of LADA by, for the first time, using a two-sample MR design. In addition, we wanted to compare these associations in LADA and type 2 diabetes.

## Methods

### Study design

This was a two-sample MR study using summary statistics from two separate genome-wide association studies (GWAS) of non-overlapping samples of the same underlying population; one provided measures of the associations between IVs and the exposure and the other on the associations between IVs and the outcome [13].

### Genetic instruments

#### Birthweight

Birthweight can be affected by both fetal (own) SNPs and maternal SNPs (through intrauterine environment). To assess the direct association of birthweight determined by fetal SNPs with LADA, and to minimise potential bias from the intrauterine environment (pathway 2 in ESM Fig. 1), we used summary statistics (β coefficients and SEs) for the associations between fetus-only SNPs (SNPs acting only through fetal genome and not maternal genome) and own birthweight (see ESM Methods). IVs for birthweight were extracted from a meta-analysis of the Early Growth Genetics Consortium (EGG) and UK Biobank [14]. Information on birthweight had been collected by measurement at birth, obstetric records, medical registers, interviews with the mother, or self-report as adults in different included studies (ESM Table 1). The study identified 64 fetus-only SNPs associated with own birthweight at *p*<6.6×10^−9^ (genome-wide significance threshold revised by authors of the GWAS) in 298,140 European individuals. Among the 64 SNPs, we excluded nine located near genes [14] known to affect the occurrence of diabetes, insulin resistance or glucose regulation to reduce pleiotropy (pathway 3 in ESM Fig. 1; ESM Methods). We further excluded three SNPs located near imprinted genes (rs234864, rs6575803 and rs6026449) and three SNPs (rs7772579, rs8756 and rs1480470) in linkage disequilibrium (LD) (*r*^2^≥0.01) with other fetus-only SNPs (ESM Methods) [14]. Forty-three of the 49 remaining SNPs (ESM Table 2) were available in the GWAS dataset of LADA described below, explaining 0.996% of the variance in own birthweight.

#### Adult BMI

The primary set of IVs for BMI in adulthood was obtained from a meta-analysis including 681,275 individuals of European ancestry from the UK Biobank and the Genetic Investigation of Anthropometric Traits (GIANT) consortium [15]. Information on BMI had mainly been obtained through clinical measurements (ESM Table 1). SNPs of this meta-analysis have been used as IVs for BMI in a previous MR study [16]. In the meta-analysis, independent SNPs were selected through LD clumping, in which SNPs with LD measure of *r*^2^>0.01 within a 10,000 kb window were pruned, resulting in a total of 839 independent SNPs with *p*<1×10^−8^ (genome-wide significance threshold revised by authors of the meta-analysis). Of these, 817 SNPs were available in the GWAS of LADA and proxy SNPs in LD (*r*^2^>0.8) with three of the unavailable SNPs were identified using LDlink (accessed 12 March 2021) [17]. The 820 SNPs explained 7.43% of the variance in adult BMI (ESM Table 3).

The GIANT consortium had samples overlapping with the GWAS of type 2 diabetes (described below). Therefore, we selected a secondary set of IVs (734 independent SNPs) for adult BMI exclusively from the UK Biobank study (https://gwas.mrcieu.ac.uk/, accessed 3 April 2021) when comparing the BMI–LADA association with the BMI–type 2 diabetes association. The 734 SNPs explained 7.86% of the variance in adult BMI.

### GWAS of LADA

LADA was the primary outcome in the present study. We obtained summary statistics for the association between the aforementioned SNPs and LADA from the only GWAS of LADA hitherto. This study included 2634 LADA cases and 5947 population controls, comprising individuals of European ancestry from Sweden, Denmark, Germany and the UK, and the analysis was adjusted for sex and principal components (to correct for potential bias due to population structure) [1]. LADA was defined based on the following criteria: (1) adult-onset (age at diagnosis >20, 30 or 35 years); (2) the presence of diabetes-associated autoimmune autoantibodies, in particular GAD autoantibody (GADA) positivity; and (3) lack of insulin requirement for 6 months or 1 year after diagnosis [1].

### GWAS of type 2 diabetes

Summary statistics for the SNP–type 2 diabetes association were obtained from the DIAbetes Genetics Replication And Meta-analysis (DIAGRAM) Consortium, which included 26,676 type 2 diabetes cases and 132,532 controls among individuals of European ancestry [18]. Summary statistics in this study were adjusted for age, sex, and principal components [18].

### Data harmonisation

We checked the effect allele, reference allele and effect allele frequency in the GWAS datasets. The β coefficient for the SNP–exposure association was ‘flipped’ if the effect allele for the SNP–exposure association was the reference allele for the SNP–outcome association. We paid attention to palindromic (A/T or C/G) SNPs and there was no ambiguity in matching effect alleles for these SNPs between GWAS datasets of the exposure and the outcome.

### Statistical analysis

We measured instrument strength of each SNP using *F* statistic [19], which equals the square of the β coefficient for the SNP–exposure association divided by the square of the SE for the β coefficient. A larger *F* statistic indicated stronger instrument strength [19]. Birthweight and adult BMI were all inverse-normally transformed and analysed in an additive model in the GWAS. Therefore, the risk estimates based directly on the summary statistics were ORs and 95% CIs for LADA and type 2 diabetes per SD change in exposures.

#### Main analysis

The inverse-variance weighted (IVW) method was used to assess the risk of LADA in relation to birthweight based on 43 IVs and adult BMI based on 820 primary IVs. The IVW method can be fitted in weighted linear regression [20, 21]. It provides a more precise risk estimate than other methods when all the IVs are valid [22].

#### Sensitivity analyses

Central assumptions in an MR study are that an IV only affects the outcome through the exposure, not through a direct pathway to the outcome or via a confounder (ESM Fig. 2) [23], otherwise there will be directional pleiotropy and the IV is invalid.

The IVW estimator used in the main analyses assumes that all the IVs are valid [24]. Several sensitivity analyses using other MR estimators were conducted to test the robustness of the results based on the IVW estimator. Some of them can detect potential directional pleiotropy, and some of them do not require all IVs to be valid. First, we used the robust IVW method, which replaces the standard linear regression in the IVW method with a robust regression [25]. This method has a greater power than IVW to reject causal null hypothesis when there is balanced pleiotropy (average pleiotropic effect: 0) and the instrument strength is independent of the instrument’s direct effect (InSIDE [26] assumption) [24]. Furthermore, we used the weighted median method, which provides consistent estimates when >50% of IVs are valid and does not rely on the InSIDE assumption [27]. Egger regression of MR (MR-Egger) can give a causal estimate under the InSIDE [26] assumption even when all the IVs are invalid, with the slope coefficient of the regression model representing the logarithmic OR [26]. This method indicates overall directional pleiotropy [26] if the estimated intercept term in the regression model is non-zero [26]. Finally, the MR pleiotropy residual sum and outlier (MR-PRESSO) approach estimator was used. The MR-PRESSO approach is based on the IVW method and detects outliers (potential pleiotropic SNPs), produces corrected ORs by removing outliers, and evaluates distortion of risk estimate by outliers [28].

In the present study, we further excluded outliers detected by MR-PRESSO from the IVs, and then re-analysed the data using all different MR estimators including the IVW estimator.

We did other sensitivity analyses based on the IVW method by excluding some SNPs from the 43 IVs for birthweight and 820 primary IVs for adult BMI. These sensitivity analyses included several conservative analyses to minimise the possibility that the IVs affect the outcome through a pathway outside the exposure, and a leave-one-out analysis to investigate whether the association in the main analysis would disappear by excluding any one of the IVs. In the analysis of birthweight, conservative analysis 1 excluded SNPs associated with diabetes-related traits at nominal significance level (Bonferroni-corrected), to minimise the possibility that IVs affect LADA directly. We further excluded SNPs associated with any trait (except birthweight) at genome-wide significance in conservative analysis 2. In the analysis of adult BMI, we excluded SNPs associated with diabetes-related traits at nominal significance level (Bonferroni-corrected) or any trait (except adult body size) at genome-wide significance (conservative analysis 1), SNPs associated with lifestyle factors at nominal significance level (Bonferroni-corrected, conservative analysis 2), and SNPs associated with birthweight at nominal significance level (Bonferroni-corrected, conservative analysis 3). Further details about conservative analyses are provided in ESM Fig. 3 and ESM Table 4.

We additionally did a sensitivity analysis by using summary statistics adjusted for maternal genotypes for birthweight based on the 43 fetus-only SNPs [14].

#### Comparison between LADA and type 2 diabetes

For comparison between LADA and type 2 diabetes, we used the same 43 IVs for birthweight and 734 secondary IVs for adult BMI in the analyses, to ensure the comparability of results. The associations of birthweight and adult BMI with type 2 diabetes were assessed using all the different MR estimators described above. Outlier-corrected ORs (95% CIs) from MR-PRESSO were used for comparison between LADA and type 2 diabetes if MR-PRESSO detected outliers, otherwise the results of IVW were used for comparison.

MR analysis was conducted using MendelianRandomization and MR-PRESSO package in R 4.0.4 [29]. All statistical tests were two-sided, with *p*<0.05 indicating statistical significance.

### Ethical approval

No ethical permit was required as we used only GWAS summary statistics and no individual-level data.

## Results

**Fig. 1:**
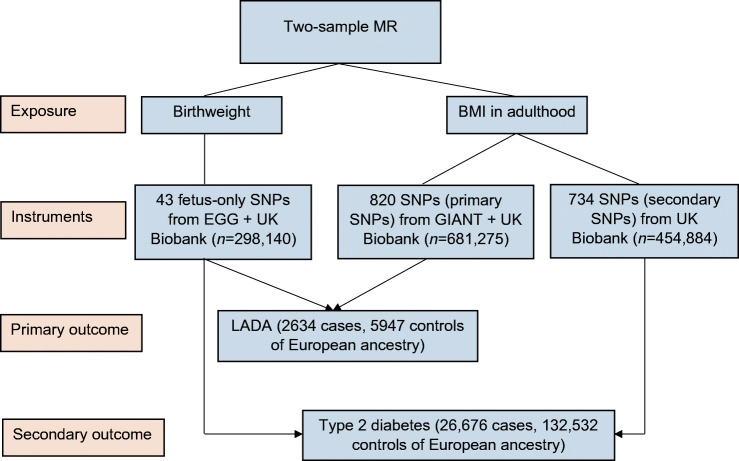
Flow chart of the design of the present study

A flow chart of the study design is provided in Fig. 1. The SD of birthweight and BMI in adulthood was approximately 500 g and 4.8 kg/m^2^, respectively (ESM Table 1). *F* statistics for IVs were all above 10 in the present study (ESM Tables 2, 3).

### Birthweight and LADA

**Fig. 2:**
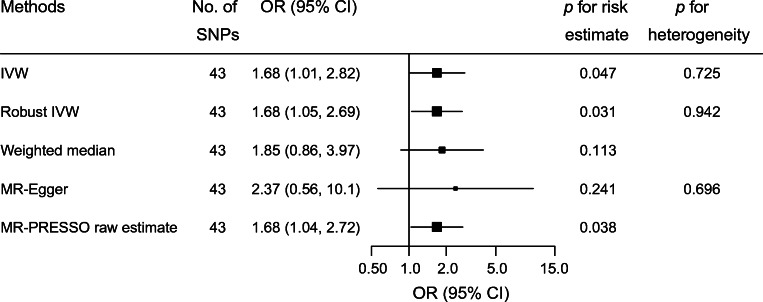
The risk of LADA in relation to one SD (500 g) decrease in birthweight. MR-Egger intercept −0.009, *p* for directional pleiotropy = 0.618. MR-PRESSO detected no outliers (*p* for global test of pleiotropy = 0.736) and the raw estimate is presented

Genetically determined birthweight based on 43 IVs was inversely associated with LADA (ESM Fig. 4). In the main analysis, the OR of LADA was 1.68 (95% CI 1.01, 2.82) for each SD decrease in birthweight (Fig. 2).

The inverse association between birthweight and LADA was also observed using other MR estimators (Fig. 2). MR-Egger indicated no directional pleiotropy (intercept −0.009, *p* for directional pleiotropy = 0.618). MR-PRESSO detected no outliers and the raw estimate was 1.68 (95% CI 1.04, 2.72).

The inverse association between birthweight and LADA was also observed in a series of conservative analyses by excluding SNPs associated with diabetes-related traits at *p*<0.05/43, and by further excluding SNPs associated with any other trait at *p*<5×10^−8^ (ESM Table 5). Leaving out one of the 43 SNPs each time did not change the direction of association, although some 95% CIs crossed 1.00 (ESM Fig. 5). Among the 43 SNPs, rs4144829 was in LD with a SNP acting through both fetal and maternal genome (rs2174633, not used as an IV in this study). The leave-one-out analysis showed that there was no major change in point estimate (1.67 [95% CI 0.99, 2.83]) after leaving rs4144829 out. The OR (1.58 [95% CI 0.96, 2.48]) estimated by using summary statistics adjusted for maternal genotypes was similar to that of the main analysis.

### Adult BMI and LADA

**Fig. 3:**
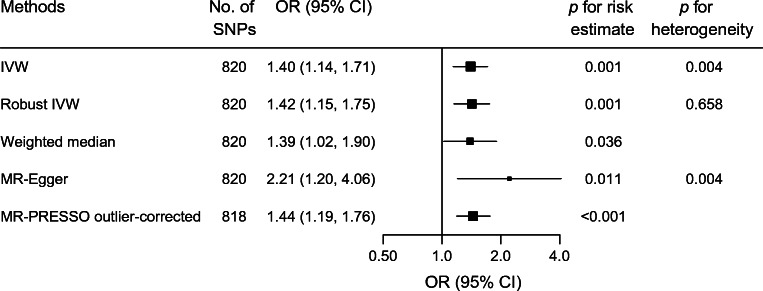
The risk of LADA in relation to one SD (4.8 kg/m^2^) increase in adult BMI. MR-PRESSO identified two outliers: rs11066188 and rs10840606. Outliers were excluded from the outlier-corrected estimate (*p* for distortion of estimate 0.777)

Genetically determined adult BMI based on the 820 primary IVs was positively associated with LADA (ESM Fig. 6). One SD increase in adult BMI was associated with an OR of 1.40 (95% CI 1.14, 1.71) for LADA using the IVW method (Fig. 3).

MR-Egger indicated no directional pleiotropy (intercept −0.007, *p* for directional pleiotropy = 0.119), while MR-PRESSO detected two outliers (rs11066188 and rs10840606). The outliers did not distort the results (*p* for distortion test = 0.777) and the outlier-corrected OR was similar to the OR obtained with IVW. Robust IVW and weighted median also showed results comparable with those estimated by IVW (Fig. 3). After excluding rs11066188 and rs10840606 from the IVs, no major change in results from different MR estimators was observed (ESM Fig. 7).

All the three conservative analyses (ESM Table 5) and the leave-one-out analysis (data not shown) showed similar results to the main analysis.

### Comparison between LADA and type 2 diabetes

When assessing the association between birthweight and type 2 diabetes, MR-PRESSO detected three outliers and indicated no distortion by outliers (*p* for distortion = 0.533; ESM Table 6). After correction for outliers, the OR for type 2 diabetes in relation to one SD decrease in genetically determined birthweight was 1.64 (95% CI 1.32, 2.04), which was similar to the magnitude of the birthweight–LADA association (Table 1). The leave-one-out analysis (ESM Fig. 8) and the sensitivity analysis using summary statistics adjusted for maternal genotypes (OR 1.47 [95% CI 1.11, 1.95]) also showed an inverse association between birthweight and type 2 diabetes.

**Table 1:** Comparison between LADA and type 2 diabetes

Exposures	Outcomes	No. of initial SNPs	No. of outliers^a^	GWAS dataset for SNP–exposure association	Methods	OR (95% CI)^b^	*p* for risk estimate
Birthweight	LADA	43	0	EGG+UK Biobank	IVW^c^	1.68 (1.01, 2.82)	0.047
Birthweight	Type 2 diabetes	43	3	EGG+UK Biobank	MR-PRESSO outlier-corrected	1.64 (1.32, 2.04)	<0.001
BMI in adulthood	LADA	734	3	UK Biobank	MR-PRESSO outlier-corrected	1.42 (1.18, 1.72)	<0.001
BMI in adulthood	Type 2 diabetes	734	9	UK Biobank	MR-PRESSO outlier-corrected	2.33 (2.16, 2.51)	<0.001

^a^Outliers were excluded from the outlier-corrected estimates

^b^OR (95% CI) for LADA or type 2 diabetes per SD (500 g) decrease in birthweight or per SD (4.8 kg/m^2^) increase in BMI in adulthood

^c^MR-PRESSO detected no outliers and therefore the result of the IVW method was presented

MR-PRESSO detected nine outliers when assessing the association between adult BMI and type 2 diabetes based on the 734 secondary IVs (ESM Table 7). The OR of type 2 diabetes was 2.33 (95% CI 2.16, 2.51) for each SD increase in adult BMI after outlier removal (Table 1 and ESM Table 7). For LADA, the same IVs yielded an outlier-corrected OR of 1.42 (95% CI 1.18, 1.72) (Table 1 and ESM Table 7), which was similar to the association between LADA and adult BMI observed based on the 820 primary IVs.

## Discussion

### Main findings

This study provides genetic evidence to show that low birthweight and adult adiposity confer an increased risk of LADA. We also confirm findings of previous MR studies showing that lower birthweight [30–32] and higher BMI [33–40] in adulthood are associated with increased risk of type 2 diabetes. To the best of our knowledge, this is the first MR study to explore the role of environmental/lifestyle factors in the aetiology of LADA.

### Main findings in relation to previous studies

The results regarding birthweight are in line with those of our previous observational study indicating a twofold increased risk of LADA in individuals with a birthweight <3 kg compared with ≥4 kg [9]. Notably, the association with birthweight was not weaker for LADA than for type 2 diabetes, also in line with previous observational data [9]. The mechanism linking birthweight to LADA and type 2 diabetes remains unclear. The Barker hypothesis proposes that an adverse intrauterine environment leads to both lower birthweight and higher risk of future cardiometabolic risk [41]. This hypothesis was not tested in our study since such analysis would require data on mother–child pairs for both the exposure and the outcome [41]. However, we restricted the IVs to SNPs with fetus-only effects; this implies that the inverse association is not explained by pleiotropy introduced by the intrauterine environment, although we cannot fully rule out the possibility that some of the fetus-only SNPs might be found to have both fetal and maternal components by larger GWAS in the future. Notably, results of our sensitivity analysis where we adjusted for maternal genotypes were also compatible with inverse associations between birthweight and LADA/type 2 diabetes. The fetal insulin hypothesis proposes that genetically determined insulin resistance in the fetus results in impaired insulin-mediated fetal growth as well as insulin resistance in adult life [42]. However, our findings do not support this hypothesis since the association between birthweight and LADA was not attenuated after excluding SNPs currently known to be associated with diabetes-related traits (including insulin resistance) at nominal significance level (Bonferroni-corrected). Our findings suggest that lower birthweight determined by own SNPs might have a causal effect on the risk of LADA. The results have public health implication in identifying individuals (those with lower birthweight) susceptible to LADA. These individuals might need to adopt a healthier lifestyle to alleviate the risk of LADA since our previous findings suggest that the combination of low birthweight and being overweight as an adult may be particularly detrimental [9]. However, we acknowledge that it is complicated to study birthweight as an exposure in an MR framework and the underlying mechanism for the suggested inverse association remains to be explored. With the accumulation of GWAS data, future MR studies based on mother–child pairs [41] are needed to gain deeper insights into the potential mechanisms linking perinatal factors to adult-onset diseases such as LADA.

Findings regarding LADA and adult BMI were also in line with previous observational data [8]. Overweight and obese status is strongly associated with development of insulin resistance [43] and this may explain a causal link between adult adiposity and LADA. In support hereof, a positive association between BMI and insulin resistance was observed in individuals with LADA [8]. The association with BMI was stronger for type 2 diabetes than LADA, which is in line with previous findings [8]. This is to be expected since insulin resistance tends to be less pronounced in LADA compared with type 2 diabetes [2, 8, 44]. A previous MR study also found support for a link between childhood adiposity and type 1 diabetes [45]. This implies that overweight/obese status is implicated in the promotion of all major types of diabetes and emphasises that it is crucial to prevent overweight status in order to reduce the incidence of diabetes.

### Assessment of MR assumptions

A major concern in MR studies is the violation of IV assumptions. These assumptions cannot be fully tested, although we used several approaches to minimise this potential bias. First, we applied different MR estimators, some of which detected and corrected for potential directional pleiotropy from a statistical perspective. There was no major change in ORs after excluding outliers, indicating the robustness of the results. Second, we excluded some SNPs in several conservative analyses, in which the associations of birthweight and adult BMI with LADA persisted. The risk of LADA is linked to genes in the HLA region, primarily susceptibility within HLA-DRB1 and HLA-DQB1 haplotypes [1, 46, 47]. Among the 43 SNPs for birthweight, no SNPs are located within 300 kb windows of HLA-related genes [14]. It should be noted that excluding SNPs in the conservative analyses does not prove that they are in fact invalid SNPs.

### Strengths and limitations

There are several strengths in the present study. First, the two-sample MR design using genetic variants as unbiased proxy minimises confounding and reverse causation. Second, the application of different MR estimators and a series of conservative analyses reduces the risk of bias caused by directional pleiotropy. Third, we confirmed findings from previous studies on type 2 diabetes and used type 2 diabetes as a ‘positive control’ to show that the instruments and methods used for LADA in the present study are reliable. Further, this provides a good opportunity to compare the aetiology of LADA and type 2 diabetes. There are also some limitations. First, the lack of individual data rules out the possibility of exploring potential non-linear association between exposures and outcomes; this is a common limitation in MR studies based on summary statistics. The linear assumption is less likely to be violated in our MR study. For LADA, there is no evidence with sufficient statistical power to support a non-linear association [8, 9]. Adult BMI seemed to be linearly (positively) associated with type 2 diabetes [35, 48]. Observational studies found that the linear (inverse) association between birthweight and type 2 diabetes held when birthweight was <4.0 kg [49] or <4.5 kg [50]. The range of birthweight (study-specific, 2.5–4.5 kg, or within the range of mean ± 5SD) in the GWAS [14] used by the present MR analysis is generally in the range of linear association. Moreover, deviation from the linear assumption is likely to reduce the statistical power in risk estimate, rather than generating spurious associations [51]. Second, findings from the present study are only applicable to the European population since the only GWAS on LADA was conducted in individuals of European ancestry. It is unclear to what extent the findings are generalisable to other populations.

## Conclusions

These findings provide genetic support for a causal link between low birthweight, adult overweight/obese status and LADA. The results persisted in a series of sensitivity analyses. Measures should be taken to reduce the prevalence of adult overweight/obese status for the prevention of diabetes with and without an autoimmune component. The mechanism linking low birthweight to diabetes remains to be explored.

## Supplementary information

ESM 1 (PDF 1.97 mb)

## Data Availability

This study only used summary data and these data are publicly available.

## Abbreviations

DIAGRAM: DIAbetes Genetics Replication And Meta-analysis Consortium
EGG: Early Growth Genetics Consortium
GIANT: Genetic Investigation of Anthropometric Traits Consortium
GWAS: Genome-wide association studies
InSIDE: Instrument Strength Independent of Direct Effect
IV: Instrumental variable
IVW: Inverse-variance weighted
LADA: Latent autoimmune diabetes in adults
LADY: Latent autoimmune diabetes in the young
MR: Mendelian randomisation
MR-Egger: Egger regression of MR
MR-PRESSO: MR pleiotropy residual sum and outlier approach
