# Supplementary material for: Birthweight, BMI in adulthood and latent autoimmune diabetes in adults: a Mendelian randomisation study
Source: Diabetologia. 2022 May 23;65(9):1510–8. doi: 10.1007/s00125-022-05725-2 (PMC9345833; doi:10.1007/s00125-022-05725-2)
Supplement: Supplementary file 1 — (PDF 1.97 mb) [file 125_2022_5725_MOESM1_ESM.pdf]

ESM Methods: Selection of SNPs and summary statistics for birthweight

Among the 64 fetus-only SNPs identified in the meta-analysis of the Early Growth Genetics Consortium (EGG) and UK Biobank[1], we excluded 9 SNPs located near genes known to affect the occurrence of diabetes, insulin resistance or glucose regulation (rs12401656 near SLC2A1, rs10181515 near IRS1, rs138715366 near GCK, rs1323438 near PAPPA, rs11042596 near INS-IGF2, rs222857 near SLC2A4, rs8106042 near INSR, rs1129156 near AKT2, and rs1012167 near MAFB)[1], 3 SNPs located near imprinted genes (rs234864 near KCNQ1, rs6575803 near DLK1 and rs6026449 near GNAS)[1, 2] and 3 SNPs in LD ($r^2 \geq 0.01$) with other fetus-only SNPs (rs7772579 in LD with rs10872678; rs8756 and rs1480470 in LD with rs7968682). Forty-three of the 49 remaining SNPs were available in the GWAS dataset of LADA, explaining 0.996% of the variance in own birthweight. Since these SNPs have been identified to affect only own birthweight but not offspring birthweight, we used summary statistics for the associations between the 43 SNPs and own birthweight unadjusted for maternal genotypes in the main analysis.

ESM Table 1. Basic characteristics of included GWAS studies for exposures

Year, author	Studies	Sample size	Ancestry	Exposure	Exposure measurement	SD of exposure	Exposure transformation	Quality control of SNPs				Adjustment
								Call rate	INFO score	MAF	HWE *P*	
2019, Warrington NM [1]	EGG + UK Biobank	298,140	European	birthweight	measurements at birth, obstetric records, medical registers, interviews with the mother, or self-report as adults	~500 g	z-score transformed in men and women separately	study-specific: from >0.9 to >0.98	unknown	study-specific: from >0.1% to >2%	study-specific: from >5e-8 to >1e-4	study-specifc covariates such as PCs
2018, Yengo L[3]	UK Biobank + GIANT	681,275 (456,426 from UK Biobank)	European	adult BMI	mostly through measurement, some through self-report	~4.8 kg/m^2	inverse-normally transformerad	GIANT: study-specific UK Biobank: >0.95	GIANT: study-specific; UK Biobank: >0.3	GIANT: study-specific; UK Biobank: >0.01%	GIANT: study-specific; UK Biobank: > 1e-6	UK Biobank: age, sex, recruitment centre, genotyping batches and 10 PCs. GIANT: age, age squared, and any necessary study-specific covariates
2018, Elsworth B	UK Biobank [a]	454,884	European	adult BMI	measured during the initial Assessment Centre visit	~4.8 kg/m^2	inverse rank-normal transformed	unknown	>0.8	>0.1%	> 1e-10	age, sex, and PCs

GWAS: genome-wide association study; MAF: major allele frequency; HWE: Hardy–Weinberg test; EGG: Early Growth Genetics Consortium; GIANT: Genetic Investigation of ANthropometric Traits consortium; PC: principal component.

[a] Data were obtained from https://gwas.mrcieu.ac.uk/datasets/ukb-b-2303/.

ESM Table 2. Detailed information on 43 instrumental variables for the association between birthweight and LADA

SNP	Effect allele	Other allele	SNP-birthweight association					SNP-LADA association		
			BETA	SE	*P*	R^2	F statistics	BETA	SE	*P*
rs10221267	T	C	0.0167	0.0026	6.5E-11	1.4E-04	42.7	-0.0177	0.0372	0.635
rs10265057	G	A	0.0273	0.0045	1.3E-09	1.2E-04	36.9	0.0838	0.0653	0.202
rs10872678	T	C	0.0317	0.0028	9.8E-29	4.0E-04	123.9	-0.0717	0.0412	0.084
rs10935733	T	C	0.0194	0.0026	2.3E-13	1.8E-04	53.8	0.0152	0.0382	0.693
rs11055030	G	C	0.0200	0.0029	3.9E-12	1.6E-04	48.3	0.0551	0.0426	0.198
rs112139215	A	C	0.0475	0.0051	2.8E-20	2.8E-04	85.2	-0.0653	0.0725	0.371
rs11698914	C	G	0.0319	0.0031	1.2E-24	3.6E-04	105.2	-0.0334	0.0435	0.445
rs11711420	T	G	0.0187	0.0030	3.2E-10	1.3E-04	39.6	0.0006	0.0438	0.989
rs11867479	T	C	0.0172	0.0027	1.1E-10	1.4E-04	41.7	0.0133	0.0389	0.733
rs13266210	A	G	0.0268	0.0031	1.5E-17	2.4E-04	72.8	0.0664	0.0467	0.157
rs134594	C	T	0.0168	0.0027	5.8E-10	1.3E-04	38.5	-0.0321	0.0394	0.418
rs147957154	T	C	0.0232	0.0039	2.8E-09	1.2E-04	35.4	0.0466	0.0551	0.400
rs1482852	A	G	0.0504	0.0026	1.6E-82	1.2E-03	370.5	-0.0312	0.0376	0.410
rs1547669	G	A	0.0178	0.0026	6.2E-12	1.6E-04	47.3	0.0049	0.0373	0.896
rs2282978	C	T	0.0183	0.0027	1.7E-11	1.5E-04	45.4	0.0059	0.0395	0.882
rs2551347	T	C	0.0245	0.0030	1.9E-16	2.2E-04	67.8	-0.0097	0.0433	0.823
rs28457693	G	A	0.0442	0.0042	9.9E-26	3.8E-04	110.1	0.0002	0.0577	0.997
rs28505901	A	G	0.0244	0.0031	2.5E-15	2.2E-04	62.8	-0.0237	0.0429	0.583
rs34036147	T	C	0.0185	0.0028	8.4E-11	1.5E-04	42.2	-0.0555	0.0397	0.165
rs351930	T	A	0.0192	0.0032	2.9E-09	1.2E-04	35.3	-0.0127	0.0472	0.790
rs3933326	G	A	0.0213	0.0028	2.3E-14	2.0E-04	58.3	-0.0194	0.0390	0.620
rs40434	G	A	0.0167	0.0027	3.0E-10	1.3E-04	39.7	-0.0224	0.0373	0.551
rs41311445	A	C	0.0326	0.0045	3.3E-13	1.9E-04	53.1	-0.1106	0.0609	0.071
rs4144829	C	T	0.0355	0.0029	4.3E-34	4.9E-04	148.4	-0.0238	0.0420	0.572
rs4444073	A	C	0.0202	0.0026	2.7E-15	2.0E-04	62.6	0.0776	0.0374	0.039
rs4511593	T	C	0.0175	0.0027	1.1E-10	1.4E-04	41.7	0.0099	0.0392	0.801
rs4953353	G	T	0.0179	0.0027	3.5E-11	1.5E-04	44.0	-0.0095	0.0389	0.809
rs61830764	A	G	0.0166	0.0027	1.1E-09	1.3E-04	37.2	0.0147	0.0383	0.703
rs6925689	T	C	0.0150	0.0026	6.4E-09	1.1E-04	33.8	-0.0300	0.0370	0.421
rs6930558	T	G	0.0218	0.0030	3.4E-13	1.8E-04	53.0	0.0160	0.0428	0.711
rs7076938	T	C	0.0321	0.0029	2.1E-28	4.0E-04	122.3	-0.0278	0.0421	0.511
rs72656010	T	C	0.0283	0.0038	1.4E-13	1.8E-04	54.8	-0.1531	0.0547	0.005
rs7285579	C	T	0.0173	0.0029	2.7E-09	1.3E-04	35.5	0.0305	0.0418	0.468
rs73143584	A	G	0.0288	0.0043	1.8E-11	1.6E-04	45.2	0.0228	0.0607	0.709
rs732563	C	T	0.0174	0.0026	1.3E-11	1.5E-04	45.9	-0.0437	0.0372	0.242
rs7402983	A	C	0.0241	0.0027	2.6E-19	2.8E-04	80.8	-0.0611	0.0382	0.112
rs753381	T	C	0.0151	0.0026	3.4E-09	1.1E-04	35.0	0.0188	0.0376	0.619
rs754868	G	A	0.0159	0.0026	6.7E-10	1.2E-04	38.2	-0.0490	0.0381	0.201
rs7819593	C	T	0.0218	0.0030	6.2E-13	1.7E-04	51.8	-0.0099	0.0424	0.816
rs7968682	G	T	0.0418	0.0026	4.2E-60	8.7E-04	267.6	-0.0092	0.0374	0.806
rs80278614	A	G	0.0404	0.0059	6.5E-12	1.7E-04	47.3	0.0188	0.0773	0.809
rs9657468	G	T	0.0149	0.0028	7.9E-08	9.9E-05	28.9	-0.0165	0.0395	0.679
rs9909342	A	G	0.0179	0.0027	2.2E-11	1.5E-04	44.9	-0.0297	0.0384	0.442

LADA: latent autoimmune diabetes in adults.

ESM Table 3. Detailed information on 820 instrumental variables for the association between BMI in adulthood and LADA

SNP	Effect allele	Other allele	SNP-BMI association					SNP-LADA association		
			BETA	SE	*P*	R^2	F statistics	BETA	SE	*P*
rs1227244	G	A	0.0106	0.0019	1.00E-08	5.02E-05	31.1	-0.0117	0.0393	7.66E-01
rs11119208	A	G	0.0095	0.0017	1.00E-08	4.28E-05	31.2	-0.0239	0.0385	5.38E-01
rs719802	T	C	0.0101	0.0018	9.50E-09	4.81E-05	31.5	-0.0063	0.0378	8.69E-01
rs1169091	C	T	0.0113	0.002	6.90E-09	5.11E-05	31.9	-0.0239	0.0417	5.68E-01
rs1277723	A	G	0.0113	0.002	1.00E-08	4.44E-05	31.9	0.0029	0.0445	9.47E-01
rs2119753	A	G	0.0102	0.0018	6.80E-09	4.95E-05	32.1	-0.0248	0.0382	5.19E-01
rs7801551	T	C	0.0102	0.0018	9.70E-09	4.78E-05	32.1	0.0075	0.0389	8.49E-01
rs4704513	G	C	0.0125	0.0022	7.40E-09	4.63E-05	32.3	-0.0454	0.0469	3.35E-01
rs9326846	G	A	0.0108	0.0019	7.10E-09	5.00E-05	32.3	0.0097	0.0391	8.06E-01
rs9475173	A	G	0.0108	0.0019	6.60E-09	5.28E-05	32.3	-0.0253	0.0381	5.09E-01
rs1885728	A	G	0.0108	0.0019	1.00E-08	5.09E-05	32.3	-0.0546	0.0403	1.77E-01
rs2270778	C	T	0.0097	0.0017	4.30E-09	4.57E-05	32.6	-0.0116	0.0378	7.61E-01
rs10118866	T	G	0.012	0.0021	6.20E-09	5.00E-05	32.7	-0.0526	0.0446	2.41E-01
rs11089885	C	T	0.0103	0.0018	4.00E-09	5.27E-05	32.7	-0.0276	0.0378	4.68E-01
rs11790280	C	T	0.0103	0.0018	7.10E-09	5.03E-05	32.7	-0.0326	0.0387	4.03E-01
rs175165	T	G	0.0103	0.0018	5.20E-09	5.07E-05	32.7	-0.0014	0.0378	9.71E-01
rs4865796	G	A	0.0103	0.0018	5.20E-09	4.53E-05	32.7	0.0109	0.0401	7.87E-01
rs1584121	G	A	0.0126	0.0022	6.00E-09	4.90E-05	32.8	0.0334	0.0464	4.74E-01
rs16833232	C	T	0.0109	0.0019	4.60E-09	5.10E-05	32.9	-0.0118	0.0401	7.70E-01
rs1020548	G	A	0.0132	0.0023	6.60E-09	4.82E-05	32.9	-0.0029	0.0496	9.54E-01
rs11649864	A	G	0.0178	0.0031	6.70E-09	5.26E-05	33.0	0.0218	0.0655	7.41E-01
rs252749	G	A	0.0115	0.002	8.20E-09	4.85E-05	33.1	0.0614	0.0440	1.65E-01
rs2832283	A	G	0.0115	0.002	5.80E-09	4.55E-05	33.1	-0.0117	0.0455	7.98E-01
rs1402025	C	T	0.0121	0.0021	3.60E-09	5.13E-05	33.2	0.0116	0.0445	7.96E-01
rs2143624	A	G	0.0098	0.0017	4.80E-09	4.47E-05	33.2	0.0356	0.0389	3.64E-01
rs3829849	T	C	0.0098	0.0017	5.90E-09	4.42E-05	33.2	-0.0109	0.0397	7.85E-01
rs9547153	G	A	0.0098	0.0017	8.70E-09	4.54E-05	33.2	0.0226	0.0383	5.57E-01
rs4430672	T	C	0.0127	0.0022	3.90E-09	5.15E-05	33.3	0.0381	0.0458	4.07E-01
rs825680	A	T	0.0104	0.0018	6.70E-09	5.26E-05	33.4	0.0326	0.0382	3.95E-01
rs535533	C	T	0.0104	0.0018	3.40E-09	5.22E-05	33.4	0.0205	0.0381	5.93E-01
rs2477017	A	G	0.0104	0.0018	5.40E-09	5.10E-05	33.4	-0.0324	0.0384	4.01E-01
rs6898812	G	T	0.0104	0.0018	4.20E-09	5.33E-05	33.4	-0.0414	0.0377	2.75E-01
rs2619976	T	C	0.0104	0.0018	6.30E-09	5.25E-05	33.4	0.0227	0.0378	5.51E-01
rs1345942	C	T	0.0104	0.0018	5.20E-09	5.05E-05	33.4	0.0710	0.0384	6.60E-02
rs2732275	A	G	0.0104	0.0018	3.80E-09	5.12E-05	33.4	0.0264	0.0380	4.89E-01
rs1608445	G	A	0.0104	0.0018	3.70E-09	5.32E-05	33.4	-0.0006	0.0377	9.87E-01
rs2195086	G	T	0.0133	0.0023	9.40E-09	4.73E-05	33.4	-0.0478	0.0515	3.55E-01
rs1117080	C	G	0.011	0.0019	7.00E-09	5.07E-05	33.5	0.0583	0.0409	1.56E-01
rs1829130	C	T	0.011	0.0019	3.70E-09	5.28E-05	33.5	0.0479	0.0403	2.37E-01
rs17105272	T	C	0.011	0.0019	3.20E-09	5.25E-05	33.5	0.0838	0.0396	3.52E-02
rs961917	C	G	0.0116	0.002	2.60E-09	5.37E-05	33.6	-0.0326	0.0412	4.31E-01
rs10732321	C	G	0.0145	0.0025	5.10E-09	5.16E-05	33.6	-0.0490	0.0525	3.53E-01
rs925421	A	G	0.0116	0.002	5.80E-09	5.25E-05	33.6	0.0035	0.0419	9.34E-01
rs7181610	A	T	0.0145	0.0025	7.80E-09	5.11E-05	33.6	0.0638	0.0556	2.54E-01
rs10263780	G	A	0.0157	0.0027	8.30E-09	5.90E-05	33.8	0.0245	0.0555	6.61E-01

SNP	Effect allele	Other allele	SNP-BMI association					SNP-LADA association		
			BETA	SE	*P*	R^2	F statistics	BETA	SE	*P*
rs3826705	C	T	0.0157	0.0027	6.60E-09	5.23E-05	33.8	-0.0396	0.0560	4.82E-01
rs4077093	T	G	0.0128	0.0022	5.10E-09	5.57E-05	33.9	0.0180	0.0459	6.97E-01
rs7694732	A	G	0.0099	0.0017	8.70E-09	4.82E-05	33.9	0.0178	0.0372	6.33E-01
rs653264	G	A	0.0099	0.0017	9.30E-09	4.88E-05	33.9	0.0220	0.0371	5.55E-01
rs4953577	T	C	0.0099	0.0017	9.90E-09	4.85E-05	33.9	0.0151	0.0376	6.89E-01
rs10942476	G	A	0.0099	0.0017	9.50E-09	4.90E-05	33.9	-0.0156	0.0369	6.75E-01
rs1158684	A	G	0.0099	0.0017	9.10E-09	4.90E-05	33.9	0.0180	0.0373	6.32E-01
rs6561710	G	A	0.0105	0.0018	2.60E-09	5.36E-05	34.0	0.0009	0.0375	9.81E-01
rs17272434	G	A	0.0105	0.0018	3.70E-09	4.73E-05	34.0	-0.0223	0.0408	5.87E-01
rs2923774	A	G	0.0105	0.0018	7.70E-09	4.96E-05	34.0	-0.0088	0.0394	8.25E-01
rs9318686	C	T	0.0105	0.0018	1.00E-08	4.93E-05	34.0	0.0084	0.0393	8.32E-01
rs6777784	T	G	0.0105	0.0018	4.70E-09	5.18E-05	34.0	0.0441	0.0388	2.58E-01
rs2269828	G	A	0.0105	0.0018	4.40E-09	4.89E-05	34.0	-0.0100	0.0397	8.02E-01
rs11926767	T	C	0.0105	0.0018	2.50E-09	5.26E-05	34.0	-0.0637	0.0381	9.63E-02
rs10757826	A	G	0.0105	0.0018	3.00E-09	4.84E-05	34.0	0.0146	0.0403	7.18E-01
rs17448885	C	G	0.0105	0.0018	7.30E-09	4.99E-05	34.0	-0.0304	0.0393	4.42E-01
rs4936671	C	G	0.0105	0.0018	6.20E-09	5.12E-05	34.0	0.0391	0.0382	3.09E-01
rs6901756	T	C	0.0146	0.0025	2.90E-09	4.57E-05	34.1	0.0081	0.0592	8.92E-01
rs7209235	G	A	0.0111	0.0019	8.00E-09	5.22E-05	34.1	0.0630	0.0413	1.29E-01
rs2973564	A	G	0.0111	0.0019	3.30E-09	5.01E-05	34.1	0.0062	0.0418	8.83E-01
rs9904177	G	A	0.0117	0.002	2.30E-09	5.44E-05	34.2	0.0822	0.0419	5.12E-02
rs9527455	C	A	0.0117	0.002	8.00E-09	4.88E-05	34.2	0.0447	0.0444	3.17E-01
rs1941213	A	C	0.0117	0.002	3.20E-09	5.48E-05	34.2	-0.0065	0.0409	8.75E-01
rs9964756	G	T	0.0164	0.0028	2.90E-09	4.98E-05	34.3	0.0076	0.0613	9.02E-01
rs17538472	T	C	0.0129	0.0022	4.20E-09	5.14E-05	34.4	-0.0148	0.0471	7.54E-01
rs2448241	G	A	0.0176	0.003	3.20E-09	5.43E-05	34.4	-0.0058	0.0607	9.24E-01
rs2304130	A	G	0.0176	0.003	2.90E-09	4.83E-05	34.4	-0.1413	0.0629	2.55E-02
rs12629015	A	G	0.0135	0.0023	2.10E-09	5.50E-05	34.5	-0.0209	0.0470	6.58E-01
rs11538	G	A	0.0135	0.0023	3.30E-09	5.39E-05	34.5	-0.0031	0.0482	9.48E-01
rs3764835	G	A	0.0141	0.0024	3.10E-09	5.15E-05	34.5	0.0433	0.0508	3.96E-01
rs6968554	G	A	0.01	0.0017	3.50E-09	4.61E-05	34.6	-0.0345	0.0392	3.81E-01
rs2489676	G	T	0.01	0.0017	8.80E-09	4.95E-05	34.6	-0.0165	0.0374	6.62E-01
rs1668633	T	C	0.01	0.0017	8.70E-09	4.89E-05	34.6	-0.0095	0.0379	8.02E-01
rs17681708	C	T	0.0106	0.0018	7.20E-09	4.83E-05	34.7	-0.0126	0.0409	7.59E-01
rs1840969	T	A	0.0106	0.0018	1.90E-09	5.43E-05	34.7	0.0061	0.0376	8.71E-01
rs9296723	C	T	0.0106	0.0018	4.00E-09	5.19E-05	34.7	-0.0702	0.0389	7.25E-02
rs17695092	T	G	0.0106	0.0018	3.40E-09	4.79E-05	34.7	0.0174	0.0399	6.64E-01
rs903959	A	T	0.0106	0.0018	1.60E-09	5.38E-05	34.7	-0.0289	0.0382	4.51E-01
rs1524277	C	T	0.0106	0.0018	2.20E-09	5.61E-05	34.7	-0.0347	0.0371	3.52E-01
rs12628891	C	T	0.0112	0.0019	3.00E-09	5.43E-05	34.7	0.0066	0.0398	8.69E-01
rs7636868	G	A	0.0112	0.0019	2.60E-09	5.35E-05	34.7	-0.0037	0.0406	9.28E-01
rs4954638	A	C	0.0118	0.002	2.90E-09	5.21E-05	34.8	0.0274	0.0443	5.39E-01
rs17056301	C	T	0.0118	0.002	2.40E-09	5.41E-05	34.8	-0.0207	0.0424	6.27E-01
rs7950748	T	A	0.0118	0.002	4.80E-09	5.29E-05	34.8	0.0435	0.0429	3.14E-01
rs6850639	T	C	0.0124	0.0021	1.80E-09	5.07E-05	34.9	0.0683	0.0470	1.48E-01
rs1956153	T	A	0.013	0.0022	6.80E-09	4.96E-05	34.9	-0.0293	0.0484	5.47E-01
rs17599948	A	G	0.013	0.0022	4.60E-09	4.70E-05	34.9	0.1029	0.0518	4.84E-02
rs16906845	G	A	0.0225	0.0038	2.20E-09	6.31E-05	35.1	0.0095	0.0742	8.99E-01

SNP	Effect allele	Other allele	SNP-BMI association					SNP-LADA association		
			BETA	SE	*P*	R^2	F statistics	BETA	SE	*P*
rs9514131	G	T	0.0154	0.0026	5.60E-09	5.03E-05	35.1	0.0611	0.0596	3.08E-01
rs833831	T	G	0.016	0.0027	2.60E-09	5.73E-05	35.1	0.0258	0.0546	6.38E-01
rs7685048	C	T	0.0101	0.0017	4.10E-09	5.08E-05	35.3	0.0072	0.0373	8.47E-01
rs1476322	A	G	0.0101	0.0017	5.00E-09	5.00E-05	35.3	0.0024	0.0375	9.49E-01
rs243387	G	A	0.0101	0.0017	4.40E-09	5.06E-05	35.3	0.0714	0.0376	5.94E-02
rs1394879	C	G	0.0101	0.0017	6.20E-09	4.96E-05	35.3	-0.0028	0.0374	9.41E-01
rs2371767	C	G	0.0107	0.0018	6.20E-09	4.58E-05	35.3	-0.0539	0.0417	1.99E-01
rs802460	T	C	0.0107	0.0018	2.80E-09	5.17E-05	35.3	0.0217	0.0388	5.78E-01
rs2527380	T	C	0.0107	0.0018	5.10E-09	5.16E-05	35.3	0.0442	0.0399	2.70E-01
rs731834	A	C	0.0107	0.0018	1.60E-09	5.72E-05	35.3	0.0308	0.0373	4.11E-01
rs10243319	T	C	0.0107	0.0018	1.20E-09	5.47E-05	35.3	-0.0157	0.0382	6.83E-01
rs7534091	G	A	0.0113	0.0019	1.00E-09	4.80E-05	35.4	-0.0082	0.0435	8.51E-01
rs1511471	A	G	0.0113	0.0019	9.90E-10	5.53E-05	35.4	0.0545	0.0400	1.76E-01
rs294704	G	T	0.0113	0.0019	4.00E-09	5.10E-05	35.4	0.0160	0.0415	7.02E-01
rs9397928	C	T	0.0119	0.002	1.40E-09	5.49E-05	35.4	0.0374	0.0410	3.65E-01
rs7117238	G	A	0.0131	0.0022	2.50E-09	4.80E-05	35.5	0.1452	0.0517	5.23E-03
rs17720922	T	C	0.0131	0.0022	2.30E-09	5.28E-05	35.5	-0.0609	0.0475	2.02E-01
rs9817583	A	G	0.0137	0.0023	2.50E-09	5.30E-05	35.5	0.0354	0.0513	4.93E-01
rs9318380	A	G	0.0143	0.0024	1.90E-09	5.40E-05	35.5	0.0734	0.0507	1.50E-01
rs17757975	T	C	0.0143	0.0024	4.20E-09	5.16E-05	35.5	0.0743	0.0560	1.87E-01
rs7770443	A	C	0.0149	0.0025	1.30E-09	5.77E-05	35.5	0.0087	0.0521	8.68E-01
rs8126575	T	G	0.0149	0.0025	4.10E-09	5.34E-05	35.5	0.1225	0.0569	3.25E-02
rs12449219	G	C	0.0155	0.0026	2.00E-09	5.64E-05	35.5	0.0309	0.0567	5.88E-01
rs12675063	T	A	0.0156	0.0026	1.30E-09	4.88E-05	36.0	0.1117	0.0595	6.19E-02
rs610634	T	C	0.0138	0.0023	1.00E-09	5.67E-05	36.0	-0.0236	0.0484	6.28E-01
rs972283	A	G	0.0096	0.0016	5.10E-09	4.60E-05	36.0	0.0319	0.0374	3.96E-01
rs252819	T	C	0.0132	0.0022	4.00E-09	5.27E-05	36.0	-0.0594	0.0491	2.30E-01
rs7652415	T	C	0.0156	0.0026	9.70E-10	5.92E-05	36.0	-0.0833	0.0547	1.30E-01
rs6561766	A	G	0.0168	0.0028	1.20E-09	5.92E-05	36.0	-0.1040	0.0559	6.46E-02
rs2035831	G	C	0.0108	0.0018	5.50E-09	5.23E-05	36.0	0.0047	0.0393	9.06E-01
rs1420341	C	T	0.0138	0.0023	2.10E-09	5.62E-05	36.0	-0.0333	0.0467	4.78E-01
rs11001963	T	C	0.0108	0.0018	1.20E-09	5.74E-05	36.0	-0.0080	0.0373	8.32E-01
rs12042959	A	G	0.0144	0.0024	3.40E-09	5.30E-05	36.0	-0.0796	0.0520	1.28E-01
rs7761673	T	A	0.0126	0.0021	1.90E-09	5.19E-05	36.0	0.0398	0.0463	3.93E-01
rs16943356	G	A	0.0198	0.0033	3.00E-09	4.96E-05	36.0	0.1384	0.0763	7.14E-02
rs11121210	C	T	0.0108	0.0018	1.30E-09	5.45E-05	36.0	-0.0678	0.0389	8.30E-02
rs3813680	A	G	0.0144	0.0024	1.60E-09	5.47E-05	36.0	0.0202	0.0513	6.96E-01
rs2836961	C	A	0.0102	0.0017	1.10E-09	4.92E-05	36.0	-0.0108	0.0379	7.77E-01
rs896183	A	G	0.0102	0.0017	2.30E-09	5.05E-05	36.0	-0.0314	0.0379	4.09E-01
rs1467693	A	T	0.0114	0.0019	1.20E-09	5.37E-05	36.0	0.0118	0.0406	7.73E-01
rs10491182	T	C	0.0187	0.0031	2.80E-09	4.71E-05	36.4	-0.0527	0.0702	4.56E-01
rs11138313	A	G	0.0169	0.0028	3.00E-09	5.07E-05	36.4	0.0593	0.0629	3.49E-01
rs11792069	A	G	0.0145	0.0024	6.50E-10	5.93E-05	36.5	-0.0570	0.0498	2.55E-01
rs8071182	A	G	0.0133	0.0022	2.10E-09	5.07E-05	36.5	-0.0069	0.0487	8.87E-01
rs248139	A	G	0.0133	0.0022	1.20E-09	5.52E-05	36.5	0.0409	0.0464	3.81E-01
rs2283093	T	C	0.0127	0.0021	3.10E-09	5.29E-05	36.6	0.0234	0.0459	6.12E-01
rs12101393	C	G	0.0127	0.0021	1.50E-09	5.53E-05	36.6	0.0371	0.0446	4.08E-01
rs1830074	C	T	0.0115	0.0019	1.40E-09	5.42E-05	36.6	-0.0252	0.0406	5.37E-01

SNP	Effect allele	Other allele	SNP-BMI association					SNP-LADA association		
			BETA	SE	*P*	R^2	F statistics	BETA	SE	*P*
rs12065553	G	A	0.0115	0.0019	8.90E-10	5.47E-05	36.6	-0.0127	0.0412	7.59E-01
rs491711	A	C	0.0115	0.0019	1.10E-09	5.72E-05	36.6	-0.0327	0.0406	4.23E-01
rs762147	G	A	0.0115	0.0019	3.30E-09	5.22E-05	36.6	0.0634	0.0429	1.41E-01
rs17120344	A	G	0.0224	0.0037	1.50E-09	5.59E-05	36.7	-0.0314	0.0804	6.98E-01
rs17019087	C	T	0.0109	0.0018	7.80E-10	5.48E-05	36.7	0.0289	0.0393	4.64E-01
rs6594967	T	C	0.0109	0.0018	3.40E-09	5.23E-05	36.7	0.0014	0.0398	9.73E-01
rs10198345	C	T	0.0109	0.0018	2.20E-09	5.24E-05	36.7	0.0195	0.0400	6.28E-01
rs9388766	T	C	0.0109	0.0018	7.60E-10	4.87E-05	36.7	-0.0218	0.0406	5.94E-01
rs12439829	T	A	0.0109	0.0018	1.10E-09	5.63E-05	36.7	-0.0085	0.0376	8.21E-01
rs13105058	T	C	0.0109	0.0018	3.10E-09	5.21E-05	36.7	-0.0427	0.0397	2.84E-01
rs11251352	G	A	0.0109	0.0018	7.00E-10	5.71E-05	36.7	0.0170	0.0380	6.57E-01
rs6819344	A	C	0.0103	0.0017	6.10E-10	5.09E-05	36.7	0.0566	0.0375	1.33E-01
rs1884389	C	T	0.0103	0.0017	4.00E-09	5.20E-05	36.7	-0.0226	0.0376	5.51E-01
rs11604688	C	T	0.0103	0.0017	3.60E-09	5.23E-05	36.7	0.1080	0.0377	4.43E-03
rs1554194	C	G	0.0103	0.0017	3.40E-09	5.29E-05	36.7	0.0386	0.0377	3.08E-01
rs9489622	A	G	0.0103	0.0017	2.80E-09	5.30E-05	36.7	-0.0326	0.0372	3.83E-01
rs4906908	G	T	0.0103	0.0017	2.50E-09	5.29E-05	36.7	-0.0403	0.0376	2.87E-01
rs138289	A	T	0.0103	0.0017	3.30E-09	5.30E-05	36.7	0.0295	0.0377	4.37E-01
rs10733051	A	G	0.0097	0.0016	2.90E-09	4.70E-05	36.8	0.0621	0.0371	9.62E-02
rs6827083	G	A	0.0097	0.0016	4.80E-09	4.61E-05	36.8	0.0095	0.0373	7.99E-01
rs11581304	C	T	0.0182	0.003	7.10E-10	5.79E-05	36.8	-0.0428	0.0664	5.21E-01
rs11577094	T	C	0.0182	0.003	6.90E-10	4.94E-05	36.8	0.0398	0.0662	5.50E-01
rs17424278	C	A	0.017	0.0028	1.90E-09	5.45E-05	36.9	0.0505	0.0614	4.14E-01
rs2279620	C	G	0.017	0.0028	1.60E-09	5.38E-05	36.9	-0.0247	0.0601	6.82E-01
rs11173522	A	C	0.0128	0.0021	1.10E-09	5.39E-05	37.2	0.0324	0.0451	4.75E-01
rs2023671	G	T	0.0122	0.002	5.80E-10	5.61E-05	37.2	-0.0335	0.0426	4.34E-01
rs16982345	A	G	0.0122	0.002	2.80E-09	5.56E-05	37.2	0.1214	0.0434	5.41E-03
rs9426003	G	A	0.0116	0.0019	1.40E-09	5.60E-05	37.3	0.0538	0.0416	1.98E-01
rs6759670	C	A	0.0116	0.0019	1.90E-09	5.37E-05	37.3	0.0249	0.0418	5.53E-01
rs2600226	C	T	0.0116	0.0019	3.70E-10	5.95E-05	37.3	0.0268	0.0393	4.99E-01
rs2155645	C	T	0.0116	0.0019	7.10E-10	5.19E-05	37.3	-0.0207	0.0427	6.30E-01
rs11754747	T	C	0.0116	0.0019	1.40E-09	5.19E-05	37.3	0.0304	0.0431	4.83E-01
rs17709991	C	T	0.011	0.0018	2.40E-09	4.75E-05	37.3	-0.0024	0.0421	9.55E-01
rs40245	A	T	0.011	0.0018	1.90E-09	5.57E-05	37.3	0.0687	0.0388	7.89E-02
rs13266989	C	G	0.011	0.0018	3.80E-10	5.81E-05	37.3	0.0116	0.0379	7.61E-01
rs4856794	G	T	0.011	0.0018	1.50E-09	5.50E-05	37.3	-0.1171	0.0395	3.19E-03
rs424539	G	C	0.011	0.0018	4.90E-10	5.75E-05	37.3	0.0159	0.0383	6.81E-01
rs7826312	C	T	0.0104	0.0017	4.90E-10	5.24E-05	37.4	0.0097	0.0379	7.99E-01
rs676749	T	A	0.0104	0.0017	1.70E-09	5.41E-05	37.4	0.0131	0.0373	7.26E-01
rs3806116	T	G	0.0104	0.0017	7.70E-10	5.11E-05	37.4	-0.0812	0.0381	3.42E-02
rs6121381	T	A	0.0147	0.0024	9.40E-10	5.60E-05	37.5	-0.0259	0.0515	6.17E-01
rs8016771	G	T	0.019	0.0031	1.10E-09	5.77E-05	37.6	-0.0395	0.0639	5.39E-01
rs8081039	T	C	0.0233	0.0038	6.10E-10	5.87E-05	37.6	-0.0230	0.0769	7.66E-01
rs6564360	G	A	0.0135	0.0022	1.20E-09	5.62E-05	37.7	0.0077	0.0476	8.72E-01
rs7674623	T	C	0.0135	0.0022	5.70E-10	5.78E-05	37.7	0.1251	0.0467	7.73E-03
rs2809395	A	T	0.0135	0.0022	2.20E-09	5.37E-05	37.7	0.0680	0.0485	1.63E-01
rs4877313	A	T	0.0129	0.0021	4.10E-10	5.68E-05	37.7	-0.0028	0.0443	9.50E-01
rs965961	A	G	0.0172	0.0028	6.90E-10	5.45E-05	37.7	0.0956	0.0636	1.35E-01

SNP	Effect allele	Other allele	SNP-BMI association					SNP-LADA association		
			BETA	SE	*P*	R^2	F statistics	BETA	SE	*P*
rs7134628	A	G	0.0172	0.0028	1.20E-09	5.45E-05	37.7	-0.0087	0.0581	8.82E-01
rs4523610	C	T	0.0123	0.002	1.30E-09	5.34E-05	37.8	-0.0109	0.0437	8.04E-01
rs17446091	C	T	0.0123	0.002	1.80E-09	4.97E-05	37.8	-0.0346	0.0458	4.52E-01
rs4518345	G	A	0.0117	0.0019	1.00E-09	5.57E-05	37.9	0.0187	0.0416	6.54E-01
rs1911746	T	C	0.0117	0.0019	1.60E-09	4.60E-05	37.9	-0.0549	0.0468	2.43E-01
rs1853639	G	A	0.0111	0.0018	1.20E-09	5.70E-05	38.0	-0.0232	0.0392	5.57E-01
rs6556301	G	T	0.0111	0.0018	4.10E-10	5.67E-05	38.0	-0.0192	0.0394	6.29E-01
rs2451746	C	G	0.0111	0.0018	4.60E-10	5.74E-05	38.0	-0.0571	0.0391	1.46E-01
rs10408013	T	C	0.0111	0.0018	8.00E-10	5.08E-05	38.0	0.0124	0.0420	7.69E-01
rs11670142	G	T	0.0111	0.0018	4.70E-10	5.89E-05	38.0	0.0333	0.0380	3.83E-01
rs2429150	C	A	0.0111	0.0018	2.70E-10	5.99E-05	38.0	0.0317	0.0379	4.07E-01
rs10830452	G	A	0.0111	0.0018	2.10E-09	5.44E-05	38.0	0.0565	0.0395	1.55E-01
rs10745785	C	T	0.0111	0.0018	1.20E-09	5.47E-05	38.0	-0.0270	0.0390	4.91E-01
rs1260326	C	T	0.0105	0.0017	3.90E-10	5.30E-05	38.1	-0.0002	0.0384	9.95E-01
rs9845966	T	G	0.0105	0.0017	2.50E-10	5.46E-05	38.1	-0.0154	0.0372	6.82E-01
rs4670627	T	C	0.0105	0.0017	2.60E-10	5.43E-05	38.1	-0.0379	0.0374	3.13E-01
rs2293605	C	T	0.0167	0.0027	3.60E-10	6.34E-05	38.3	-0.0595	0.0549	2.81E-01
rs1363695	C	T	0.013	0.0021	3.30E-10	6.03E-05	38.3	-0.0724	0.0433	9.62E-02
rs7615297	C	G	0.0149	0.0024	5.70E-10	5.55E-05	38.5	0.0086	0.0541	8.74E-01
rs7600699	G	C	0.0149	0.0024	6.40E-10	5.90E-05	38.5	0.0167	0.0508	7.44E-01
rs2874800	G	A	0.0118	0.0019	2.60E-10	6.07E-05	38.6	-0.0610	0.0394	1.24E-01
rs10915840	G	A	0.0118	0.0019	1.30E-09	5.65E-05	38.6	-0.0267	0.0418	5.25E-01
rs6921533	T	C	0.0118	0.0019	5.60E-10	5.71E-05	38.6	0.0481	0.0408	2.41E-01
rs460799	G	A	0.0118	0.0019	6.40E-10	5.46E-05	38.6	-0.0096	0.0417	8.20E-01
rs6781254	T	C	0.0112	0.0018	4.90E-10	5.30E-05	38.7	0.0075	0.0407	8.54E-01
rs13250058	T	G	0.0112	0.0018	2.90E-10	5.49E-05	38.7	0.0088	0.0405	8.29E-01
rs223051	T	C	0.0112	0.0018	1.10E-09	5.50E-05	38.7	-0.0102	0.0395	7.97E-01
rs2718786	A	G	0.0112	0.0018	2.70E-10	5.92E-05	38.7	0.0785	0.0385	4.30E-02
rs1937684	A	T	0.0112	0.0018	8.70E-10	5.64E-05	38.7	0.0504	0.0395	2.05E-01
rs13298062	A	G	0.0137	0.0022	3.80E-10	6.05E-05	38.8	0.0264	0.0463	5.71E-01
rs1455137	C	A	0.0106	0.0017	4.40E-10	5.30E-05	38.9	0.0218	0.0380	5.68E-01
rs2317299	T	C	0.0106	0.0017	1.30E-09	5.54E-05	38.9	0.0472	0.0374	2.09E-01
rs223391	G	A	0.0131	0.0021	1.60E-10	5.38E-05	38.9	-0.0075	0.0462	8.72E-01
rs213533	C	A	0.0156	0.0025	4.80E-10	5.96E-05	38.9	0.0221	0.0549	6.89E-01
rs1465900	A	C	0.0125	0.002	4.80E-10	5.34E-05	39.1	0.0358	0.0446	4.26E-01
rs3769948	A	G	0.0125	0.002	2.90E-10	5.81E-05	39.1	0.0505	0.0419	2.31E-01
rs4911442	A	G	0.015	0.0024	5.40E-10	5.00E-05	39.1	-0.0212	0.0582	7.18E-01
rs17619973	A	G	0.02	0.0032	2.40E-10	5.52E-05	39.1	0.0215	0.0717	7.66E-01
rs420158	C	T	0.0119	0.0019	2.10E-10	5.53E-05	39.2	0.0073	0.0428	8.66E-01
rs7123876	C	T	0.0119	0.0019	2.20E-10	5.25E-05	39.2	0.0852	0.0428	4.79E-02
rs7929418	T	C	0.0113	0.0018	1.70E-10	6.06E-05	39.4	-0.0059	0.0380	8.78E-01
rs1899898	T	C	0.0113	0.0018	6.80E-10	5.56E-05	39.4	-0.0129	0.0402	7.50E-01
rs17573940	G	A	0.0113	0.0018	8.00E-10	5.28E-05	39.4	0.0138	0.0412	7.39E-01
rs10989568	A	G	0.0107	0.0017	6.60E-10	5.69E-05	39.6	0.0170	0.0376	6.52E-01
rs621042	C	A	0.0107	0.0017	6.30E-10	5.67E-05	39.6	-0.0152	0.0374	6.86E-01
rs2198679	A	G	0.0107	0.0017	5.60E-10	5.70E-05	39.6	0.0131	0.0373	7.27E-01
rs2450444	G	A	0.0107	0.0017	3.70E-10	5.20E-05	39.6	0.0254	0.0394	5.21E-01
rs702820	C	T	0.017	0.0027	1.90E-10	5.91E-05	39.6	0.0099	0.0580	8.66E-01

SNP	Effect allele	Other allele	SNP-BMI association					SNP-LADA association		
			BETA	SE	*P*	R^2	F statistics	BETA	SE	*P*
rs1304070	A	G	0.0126	0.002	3.70E-10	5.77E-05	39.7	-0.0324	0.0436	4.60E-01
rs459552	T	A	0.0126	0.002	1.70E-10	5.64E-05	39.7	0.0409	0.0442	3.57E-01
rs17450772	C	T	0.0189	0.003	2.90E-10	7.13E-05	39.7	-0.0522	0.0595	3.83E-01
rs9806058	A	T	0.0164	0.0026	4.00E-10	5.80E-05	39.8	-0.0067	0.0566	9.07E-01
rs9848399	A	G	0.0164	0.0026	2.10E-10	5.87E-05	39.8	0.0187	0.0552	7.37E-01
rs4916229	G	C	0.0183	0.0029	4.90E-10	5.63E-05	39.8	0.0003	0.0628	9.96E-01
rs11118308	A	G	0.0101	0.0016	4.80E-10	5.08E-05	39.8	0.0014	0.0374	9.71E-01
rs249292	T	C	0.012	0.0019	1.90E-10	6.17E-05	39.9	0.0611	0.0414	1.42E-01
rs774211	T	C	0.0139	0.0022	4.60E-10	5.11E-05	39.9	0.0050	0.0518	9.24E-01
rs4722398	T	C	0.0158	0.0025	3.60E-10	5.78E-05	39.9	-0.0132	0.0542	8.08E-01
rs7865157	T	C	0.0177	0.0028	3.70E-10	5.98E-05	40.0	-0.0562	0.0589	3.43E-01
rs17608150	T	C	0.0196	0.0031	1.30E-10	5.35E-05	40.0	-0.0121	0.0713	8.66E-01
rs2837398	C	A	0.0114	0.0018	1.10E-10	6.26E-05	40.1	-0.0733	0.0377	5.35E-02
rs4814512	A	C	0.0133	0.0021	2.30E-10	6.03E-05	40.1	0.0657	0.0444	1.41E-01
rs4663629	A	G	0.0133	0.0021	1.10E-10	6.42E-05	40.1	0.0249	0.0440	5.74E-01
rs10163018	C	T	0.0114	0.0018	1.50E-10	6.09E-05	40.1	0.0112	0.0383	7.71E-01
rs13201877	G	A	0.0152	0.0024	2.70E-10	5.56E-05	40.1	-0.0025	0.0543	9.63E-01
rs16940823	C	A	0.0146	0.0023	9.00E-11	6.57E-05	40.3	0.0217	0.0471	6.48E-01
rs779206	G	A	0.0127	0.002	1.10E-10	6.30E-05	40.3	0.0265	0.0425	5.35E-01
rs9463175	C	T	0.0108	0.0017	4.20E-10	5.30E-05	40.4	0.0309	0.0387	4.27E-01
rs6852276	G	A	0.0108	0.0017	8.50E-11	5.61E-05	40.4	0.0689	0.0377	6.93E-02
rs11577179	G	A	0.0108	0.0017	1.50E-10	5.45E-05	40.4	-0.0194	0.0388	6.18E-01
rs4783241	G	C	0.0108	0.0017	4.00E-10	5.83E-05	40.4	-0.0015	0.0373	9.68E-01
rs16965225	T	G	0.0216	0.0034	1.70E-10	5.87E-05	40.4	0.0954	0.0767	2.16E-01
rs10009336	C	T	0.014	0.0022	2.20E-10	5.37E-05	40.5	0.0239	0.0496	6.32E-01
rs7334078	T	C	0.0121	0.0019	2.20E-10	6.01E-05	40.6	0.0915	0.0414	2.80E-02
rs11115176	T	C	0.0121	0.0019	2.00E-10	5.34E-05	40.6	0.0133	0.0441	7.64E-01
rs2174367	G	T	0.0121	0.0019	9.30E-11	6.30E-05	40.6	-0.0063	0.0408	8.77E-01
rs7802342	G	T	0.0121	0.0019	3.10E-10	6.02E-05	40.6	-0.0166	0.0410	6.88E-01
rs2170382	T	C	0.0172	0.0027	2.40E-10	6.26E-05	40.6	-0.0408	0.0596	4.96E-01
rs3851083	G	A	0.0102	0.0016	4.10E-10	5.11E-05	40.6	-0.0254	0.0375	5.01E-01
rs17236194	C	T	0.0153	0.0024	8.30E-11	5.45E-05	40.6	0.0654	0.0544	2.32E-01
rs4072917	A	G	0.0115	0.0018	6.90E-11	6.59E-05	40.8	0.0335	0.0375	3.74E-01
rs1863652	G	A	0.0115	0.0018	1.40E-10	5.98E-05	40.8	-0.0142	0.0398	7.23E-01
rs11917965	C	A	0.0115	0.0018	7.20E-11	6.30E-05	40.8	0.0030	0.0384	9.38E-01
rs852056	T	C	0.0128	0.002	1.80E-10	6.00E-05	41.0	-0.0070	0.0441	8.74E-01
rs1865341	T	C	0.0128	0.002	2.50E-10	6.01E-05	41.0	0.0516	0.0431	2.35E-01
rs17551974	C	A	0.0141	0.0022	1.90E-10	5.82E-05	41.1	0.0551	0.0486	2.60E-01
rs12597712	G	C	0.0109	0.0017	1.40E-10	5.79E-05	41.1	-0.0135	0.0375	7.20E-01
rs7560871	A	G	0.0218	0.0034	9.60E-11	6.46E-05	41.1	0.0095	0.0721	8.96E-01
rs7805441	T	C	0.0109	0.0017	3.40E-10	5.94E-05	41.1	-0.0327	0.0375	3.86E-01
rs8036040	A	C	0.0109	0.0017	2.70E-10	5.94E-05	41.1	0.0540	0.0373	1.50E-01
rs2470520	C	T	0.0109	0.0017	5.00E-10	5.69E-05	41.1	-0.0290	0.0377	4.45E-01
rs5751239	T	C	0.0109	0.0017	3.60E-10	5.93E-05	41.1	-0.0750	0.0372	4.49E-02
rs7006629	T	C	0.0109	0.0017	2.50E-10	5.92E-05	41.1	0.0973	0.0369	8.76E-03
rs2282802	A	G	0.0109	0.0017	4.00E-10	5.87E-05	41.1	-0.0640	0.0373	8.82E-02
rs784944	A	G	0.0122	0.0019	3.90E-10	5.60E-05	41.2	-0.0566	0.0433	1.94E-01
rs1544459	C	T	0.0103	0.0016	2.20E-10	5.26E-05	41.4	-0.0655	0.0371	7.93E-02

SNP	Effect allele	Other allele	SNP-BMI association					SNP-LADA association		
			BETA	SE	*P*	R^2	F statistics	BETA	SE	*P*
rs2605603	G	A	0.0103	0.0016	2.50E-10	5.30E-05	41.4	-0.0017	0.0375	9.63E-01
rs4802778	G	A	0.0116	0.0018	8.40E-11	6.58E-05	41.5	-0.0159	0.0378	6.77E-01
rs6764533	A	G	0.0116	0.0018	1.40E-10	6.19E-05	41.5	0.0217	0.0384	5.76E-01
rs755407	T	C	0.0116	0.0018	2.30E-10	5.44E-05	41.5	0.0433	0.0412	2.97E-01
rs10803762	A	G	0.0116	0.0018	3.50E-10	5.87E-05	41.5	0.0448	0.0401	2.67E-01
rs159032	T	C	0.0129	0.002	1.90E-10	6.14E-05	41.6	0.0911	0.0423	3.23E-02
rs2815324	C	T	0.0129	0.002	1.00E-10	5.65E-05	41.6	-0.0212	0.0440	6.32E-01
rs2866816	T	C	0.0129	0.002	5.60E-11	6.41E-05	41.6	0.0121	0.0422	7.77E-01
rs12148330	A	T	0.0142	0.0022	1.50E-10	5.98E-05	41.7	-0.0436	0.0486	3.72E-01
rs2074881	C	T	0.0155	0.0024	4.90E-11	5.23E-05	41.7	0.0934	0.0520	7.44E-02
rs1523768	G	A	0.011	0.0017	2.60E-10	5.27E-05	41.9	-0.0341	0.0399	3.96E-01
rs9615905	T	C	0.011	0.0017	2.70E-10	5.99E-05	41.9	0.0163	0.0377	6.66E-01
rs331949	C	T	0.011	0.0017	1.00E-10	5.66E-05	41.9	-0.0850	0.0385	2.80E-02
rs9361779	A	C	0.011	0.0017	1.60E-10	5.97E-05	41.9	0.0605	0.0375	1.09E-01
rs7220138	G	C	0.0123	0.0019	2.70E-10	6.40E-05	41.9	-0.0922	0.0412	2.61E-02
rs11170468	A	C	0.0123	0.0019	1.90E-10	5.40E-05	41.9	-0.0193	0.0430	6.56E-01
rs5742914	T	C	0.0175	0.0027	8.80E-11	7.17E-05	42.0	0.0657	0.0545	2.31E-01
rs10058464	C	A	0.0175	0.0027	1.30E-10	6.09E-05	42.0	-0.0773	0.0603	2.03E-01
rs13432055	C	T	0.0117	0.0018	8.90E-11	5.59E-05	42.3	0.0221	0.0415	5.97E-01
rs1485038	T	C	0.0143	0.0022	7.40E-11	6.63E-05	42.3	-0.0143	0.0463	7.59E-01
rs1526665	C	T	0.0117	0.0018	4.70E-11	6.61E-05	42.3	0.0350	0.0379	3.58E-01
rs11739877	T	C	0.0117	0.0018	6.60E-11	6.50E-05	42.3	0.0309	0.0379	4.18E-01
rs4523552	T	C	0.0137	0.0021	4.00E-11	6.50E-05	42.6	-0.0001	0.0437	9.98E-01
rs4796243	G	A	0.0124	0.0019	6.10E-11	6.49E-05	42.6	-0.0683	0.0412	9.92E-02
rs4858887	C	A	0.0124	0.0019	2.10E-11	6.68E-05	42.6	-0.0882	0.0406	3.10E-02
rs10811901	A	G	0.0111	0.0017	2.50E-11	6.07E-05	42.6	-0.0167	0.0376	6.59E-01
rs1903579	C	G	0.0111	0.0017	1.10E-10	6.10E-05	42.6	0.0230	0.0373	5.39E-01
rs11075489	C	T	0.0111	0.0017	1.30E-10	6.15E-05	42.6	-0.0158	0.0374	6.75E-01
rs1399471	G	C	0.0131	0.002	2.70E-11	6.62E-05	42.9	0.0035	0.0425	9.34E-01
rs12905439	C	G	0.0118	0.0018	1.40E-10	6.24E-05	43.0	-0.0082	0.0389	8.34E-01
rs7784465	C	T	0.0164	0.0025	1.10E-10	6.45E-05	43.0	0.0531	0.0541	3.30E-01
rs7748777	A	G	0.0105	0.0016	1.60E-10	5.47E-05	43.1	0.0268	0.0376	4.78E-01
rs17001561	A	G	0.0151	0.0023	3.80E-11	6.04E-05	43.1	-0.0648	0.0521	2.16E-01
rs11753081	T	G	0.0138	0.0021	8.90E-11	5.50E-05	43.2	-0.0930	0.0479	5.37E-02
rs6023633	G	A	0.0138	0.0021	5.10E-11	6.89E-05	43.2	-0.0440	0.0439	3.18E-01
rs2616192	T	G	0.0125	0.0019	2.10E-11	6.83E-05	43.3	0.0117	0.0391	7.67E-01
rs12149756	G	A	0.0125	0.0019	3.70E-11	5.94E-05	43.3	-0.0054	0.0431	9.01E-01
rs1787267	G	C	0.0237	0.0036	3.40E-11	6.60E-05	43.3	-0.0382	0.0727	6.01E-01
rs11781222	T	C	0.0158	0.0024	5.30E-11	5.60E-05	43.3	0.0430	0.0594	4.72E-01
rs486359	C	G	0.0112	0.0017	1.60E-11	6.27E-05	43.4	-0.0090	0.0372	8.09E-01
rs7941030	C	T	0.0112	0.0017	2.00E-11	5.95E-05	43.4	-0.0429	0.0382	2.64E-01
rs823074	T	C	0.0112	0.0017	1.60E-10	6.08E-05	43.4	0.0207	0.0374	5.82E-01
rs4759075	T	C	0.0112	0.0017	1.40E-11	6.03E-05	43.4	0.0270	0.0378	4.78E-01
rs9688431	T	C	0.0231	0.0035	2.40E-11	6.05E-05	43.6	0.0257	0.0753	7.34E-01
rs2425840	C	A	0.0119	0.0018	1.60E-11	6.83E-05	43.7	-0.0178	0.0383	6.44E-01
rs6767619	C	G	0.0119	0.0018	8.10E-11	6.40E-05	43.7	-0.0002	0.0395	9.95E-01
rs1912631	G	A	0.0119	0.0018	1.30E-11	6.84E-05	43.7	-0.0536	0.0381	1.62E-01
rs1371108	A	C	0.0119	0.0018	9.00E-11	6.21E-05	43.7	-0.0296	0.0396	4.58E-01

SNP	Effect allele	Other allele	SNP-BMI association					SNP-LADA association		
			BETA	SE	*P*	R^2	F statistics	BETA	SE	*P*
rs10883553	A	C	0.0119	0.0018	1.90E-11	7.01E-05	43.7	0.0464	0.0371	2.14E-01
rs7147503	C	T	0.0119	0.0018	2.00E-11	6.60E-05	43.7	-0.0212	0.0385	5.84E-01
rs1187352	C	T	0.0119	0.0018	6.00E-11	6.43E-05	43.7	0.0112	0.0392	7.77E-01
rs6661316	T	C	0.0106	0.0016	6.80E-11	5.49E-05	43.9	-0.0501	0.0375	1.84E-01
rs7973955	G	A	0.0126	0.0019	3.70E-11	6.47E-05	44.0	-0.0300	0.0413	4.71E-01
rs8089514	A	T	0.0126	0.0019	1.10E-11	7.31E-05	44.0	0.0270	0.0391	4.93E-01
rs1006353	A	G	0.0126	0.0019	2.30E-11	5.93E-05	44.0	-0.0338	0.0423	4.26E-01
rs7249143	T	G	0.0126	0.0019	3.60E-11	6.77E-05	44.0	-0.0643	0.0413	1.21E-01
rs2543132	C	G	0.0146	0.0022	5.00E-11	6.47E-05	44.0	0.0501	0.0482	3.02E-01
rs10461497	T	C	0.0113	0.0017	5.40E-11	6.35E-05	44.2	0.0035	0.0375	9.27E-01
rs2100814	A	G	0.0113	0.0017	1.20E-10	6.18E-05	44.2	-0.0488	0.0384	2.07E-01
rs1128249	T	G	0.0113	0.0017	8.50E-12	6.13E-05	44.2	-0.0316	0.0377	4.04E-01
rs2012502	A	C	0.0113	0.0017	4.20E-11	5.96E-05	44.2	0.0715	0.0384	6.42E-02
rs4989244	G	A	0.0113	0.0017	7.50E-11	6.18E-05	44.2	0.0536	0.0376	1.57E-01
rs761423	T	C	0.0113	0.0017	5.50E-11	6.32E-05	44.2	-0.0080	0.0375	8.32E-01
rs12206564	C	T	0.0113	0.0017	5.40E-11	6.38E-05	44.2	-0.0185	0.0372	6.22E-01
rs13168288	A	G	0.0133	0.002	6.40E-11	6.64E-05	44.2	-0.0125	0.0428	7.71E-01
rs980329	C	T	0.0133	0.002	2.50E-11	6.60E-05	44.2	0.0269	0.0426	5.31E-01
rs6804181	A	T	0.0153	0.0023	5.50E-11	6.87E-05	44.3	-0.0295	0.0496	5.55E-01
rs6849518	T	C	0.0173	0.0026	2.60E-11	6.61E-05	44.3	0.0056	0.0555	9.20E-01
rs12779943	T	C	0.0213	0.0032	2.00E-11	6.84E-05	44.3	0.0382	0.0694	5.84E-01
rs2063177	A	G	0.012	0.0018	1.60E-11	6.59E-05	44.4	0.0130	0.0390	7.41E-01
rs13425435	A	C	0.012	0.0018	2.70E-11	6.39E-05	44.4	0.0386	0.0398	3.35E-01
rs1634350	A	C	0.012	0.0018	7.60E-12	7.02E-05	44.4	-0.0119	0.0375	7.53E-01
rs17776719	G	A	0.0167	0.0025	1.70E-11	6.95E-05	44.6	-0.0119	0.0526	8.23E-01
rs10438964	C	T	0.0127	0.0019	7.60E-11	6.44E-05	44.7	0.0212	0.0418	6.15E-01
rs4682718	G	A	0.0154	0.0023	9.90E-12	7.00E-05	44.8	0.0611	0.0474	2.00E-01
rs10499275	C	G	0.0154	0.0023	3.40E-11	6.54E-05	44.8	0.0117	0.0487	8.11E-01
rs3902951	G	T	0.0134	0.002	7.00E-12	6.65E-05	44.9	0.0544	0.0428	2.06E-01
rs12422552	G	C	0.0134	0.002	1.60E-11	7.02E-05	44.9	-0.0089	0.0431	8.37E-01
rs13209968	C	G	0.0114	0.0017	3.10E-11	6.49E-05	45.0	-0.0364	0.0374	3.33E-01
rs4745794	G	A	0.0114	0.0017	7.20E-11	6.49E-05	45.0	0.0811	0.0371	2.99E-02
rs1430387	T	C	0.0114	0.0017	5.80E-11	6.37E-05	45.0	0.0216	0.0378	5.71E-01
rs10768994	T	C	0.0114	0.0017	6.40E-12	6.38E-05	45.0	-0.0893	0.0376	1.84E-02
rs7683836	G	A	0.0114	0.0017	6.30E-11	6.46E-05	45.0	-0.0607	0.0377	1.09E-01
rs10818938	A	G	0.0114	0.0017	5.10E-11	6.34E-05	45.0	-0.0589	0.0375	1.18E-01
rs1658820	T	G	0.0141	0.0021	5.90E-12	7.39E-05	45.1	0.0022	0.0432	9.60E-01
rs12448738	C	A	0.0168	0.0025	2.80E-11	6.68E-05	45.2	-0.0603	0.0553	2.78E-01
rs2237403	C	T	0.0121	0.0018	3.30E-11	6.59E-05	45.2	-0.0634	0.0391	1.07E-01
rs9299	T	C	0.0121	0.0018	3.60E-11	6.69E-05	45.2	0.1024	0.0391	9.22E-03
rs11129662	G	A	0.0121	0.0018	4.50E-11	6.49E-05	45.2	0.0845	0.0386	2.97E-02
rs820071	G	T	0.0121	0.0018	1.40E-11	6.65E-05	45.2	0.0918	0.0392	1.98E-02
rs9367368	T	C	0.0121	0.0018	1.00E-11	6.19E-05	45.2	0.0208	0.0398	6.03E-01
rs1285245	G	C	0.0121	0.0018	1.70E-11	6.80E-05	45.2	-0.0361	0.0384	3.50E-01
rs2009416	C	T	0.0121	0.0018	1.10E-11	6.75E-05	45.2	0.0103	0.0388	7.91E-01
rs962796	T	C	0.0148	0.0022	5.90E-12	7.14E-05	45.3	0.0371	0.0464	4.26E-01
rs1982441	T	G	0.0175	0.0026	7.00E-12	7.29E-05	45.3	0.0150	0.0515	7.71E-01
rs12776880	A	T	0.0128	0.0019	3.00E-11	7.10E-05	45.4	0.0285	0.0404	4.84E-01

SNP	Effect allele	Other allele	SNP-BMI association					SNP-LADA association		
			BETA	SE	*P*	R^2	F statistics	BETA	SE	*P*
rs2731277	C	T	0.0135	0.002	8.80E-12	6.90E-05	45.6	-0.0642	0.0430	1.37E-01
rs4717623	C	T	0.0135	0.002	4.20E-12	6.93E-05	45.6	-0.0434	0.0414	2.97E-01
rs1199334	A	G	0.0142	0.0021	7.30E-12	6.15E-05	45.7	0.0027	0.0481	9.56E-01
rs4307239	G	A	0.0115	0.0017	3.90E-11	6.57E-05	45.8	0.0008	0.0378	9.83E-01
rs9951893	C	T	0.0115	0.0017	3.30E-11	6.60E-05	45.8	-0.0146	0.0374	6.98E-01
rs6545709	G	A	0.0203	0.003	1.50E-11	7.35E-05	45.8	-0.0586	0.0621	3.48E-01
rs2051559	C	T	0.0176	0.0026	5.00E-12	7.04E-05	45.8	0.0222	0.0548	6.87E-01
rs6014523	T	C	0.0149	0.0022	1.40E-11	7.02E-05	45.9	0.0160	0.0480	7.40E-01
rs10883759	G	A	0.0122	0.0018	5.40E-12	6.28E-05	45.9	0.0849	0.0410	3.98E-02
rs4653017	T	C	0.0122	0.0018	4.50E-11	6.46E-05	45.9	-0.0384	0.0401	3.41E-01
rs10824218	A	T	0.0122	0.0018	7.20E-12	7.36E-05	45.9	0.0054	0.0371	8.84E-01
rs9814633	A	G	0.0122	0.0018	2.10E-11	6.71E-05	45.9	-0.0437	0.0391	2.66E-01
rs7760082	G	A	0.0122	0.0018	2.90E-11	6.64E-05	45.9	-0.0179	0.0396	6.53E-01
rs4082793	C	T	0.0122	0.0018	3.60E-12	7.26E-05	45.9	0.0300	0.0377	4.30E-01
rs7925748	G	A	0.0122	0.0018	3.30E-12	7.16E-05	45.9	-0.0034	0.0379	9.30E-01
rs2467594	G	A	0.0122	0.0018	6.50E-12	6.89E-05	45.9	0.0543	0.0386	1.62E-01
rs10975933	C	G	0.0122	0.0018	2.70E-11	6.72E-05	45.9	-0.0216	0.0393	5.86E-01
rs6676084	C	T	0.0122	0.0018	4.90E-12	6.36E-05	45.9	0.0099	0.0404	8.07E-01
rs4858193	T	C	0.0129	0.0019	1.60E-11	6.68E-05	46.1	-0.0133	0.0405	7.43E-01
rs16846136	A	C	0.0129	0.0019	3.20E-11	6.52E-05	46.1	0.0179	0.0416	6.68E-01
rs12888545	G	A	0.0136	0.002	9.10E-12	6.97E-05	46.2	0.0621	0.0423	1.45E-01
rs12922346	C	G	0.0136	0.002	1.00E-11	7.22E-05	46.2	0.0499	0.0417	2.34E-01
rs849135	A	G	0.0109	0.0016	2.00E-11	5.94E-05	46.4	-0.0775	0.0372	3.81E-02
rs6011457	T	A	0.0116	0.0017	2.70E-11	6.73E-05	46.6	0.0714	0.0371	5.59E-02
rs12476772	A	C	0.0123	0.0018	8.30E-12	6.72E-05	46.7	0.0751	0.0394	5.81E-02
rs10840606	G	A	0.0164	0.0024	3.20E-12	7.73E-05	46.7	-0.2034	0.0487	3.33E-05
rs6713781	G	C	0.0123	0.0018	3.80E-12	7.34E-05	46.7	-0.0060	0.0375	8.73E-01
rs7844647	T	C	0.0123	0.0018	2.80E-11	5.94E-05	46.7	0.0073	0.0424	8.64E-01
rs7578575	A	T	0.013	0.0019	1.90E-11	7.06E-05	46.8	0.0080	0.0417	8.50E-01
rs17789218	C	T	0.013	0.0019	7.40E-12	6.15E-05	46.8	0.0078	0.0443	8.60E-01
rs934515	A	G	0.0185	0.0027	7.60E-12	7.11E-05	46.9	-0.0149	0.0593	8.02E-01
rs3930349	C	A	0.0144	0.0021	4.20E-12	7.11E-05	47.0	0.0139	0.0452	7.59E-01
rs13063194	C	T	0.0144	0.0021	1.10E-11	6.79E-05	47.0	0.0504	0.0477	2.94E-01
rs2850969	C	T	0.0165	0.0024	1.30E-11	6.66E-05	47.3	-0.1429	0.0573	1.31E-02
rs17014375	G	T	0.0172	0.0025	1.10E-11	6.90E-05	47.3	-0.0880	0.0546	1.09E-01
rs1891215	C	T	0.0117	0.0017	1.30E-11	6.79E-05	47.4	-0.0054	0.0376	8.87E-01
rs13110266	G	A	0.0117	0.0017	1.90E-12	6.61E-05	47.4	0.0130	0.0384	7.37E-01
rs7172627	G	A	0.0117	0.0017	1.10E-11	6.82E-05	47.4	0.0641	0.0372	8.67E-02
rs1964927	G	A	0.0124	0.0018	5.00E-12	7.10E-05	47.5	-0.0509	0.0379	1.82E-01
rs263041	A	G	0.0124	0.0018	2.20E-12	7.14E-05	47.5	0.0065	0.0386	8.68E-01
rs11635675	T	G	0.0124	0.0018	1.30E-11	7.03E-05	47.5	-0.0040	0.0389	9.19E-01
rs7024334	T	G	0.0138	0.002	3.10E-12	6.66E-05	47.6	0.0708	0.0443	1.12E-01
rs7083450	T	C	0.0159	0.0023	1.70E-12	6.84E-05	47.8	0.0054	0.0502	9.15E-01
rs1409818	T	C	0.0201	0.0029	2.50E-12	8.26E-05	48.0	-0.0372	0.0596	5.35E-01
rs936227	G	A	0.0118	0.0017	2.30E-12	6.62E-05	48.2	-0.0272	0.0384	4.80E-01
rs1730859	G	A	0.0118	0.0017	1.10E-11	6.26E-05	48.2	0.0591	0.0400	1.41E-01
rs4880341	C	T	0.0118	0.0017	1.10E-11	6.86E-05	48.2	0.0317	0.0374	3.99E-01
rs1421334	A	C	0.0125	0.0018	1.00E-12	7.75E-05	48.2	0.0846	0.0374	2.47E-02

SNP	Effect allele	Other allele	SNP-BMI association					SNP-LADA association		
			BETA	SE	*P*	R^2	F statistics	BETA	SE	*P*
rs7133378	A	G	0.0125	0.0018	9.40E-13	6.88E-05	48.2	-0.0603	0.0404	1.38E-01
rs33436	G	A	0.0125	0.0018	3.00E-12	7.29E-05	48.2	-0.0883	0.0390	2.46E-02
rs7206608	G	C	0.0132	0.0019	1.30E-12	7.51E-05	48.3	-0.0269	0.0397	5.01E-01
rs1035010	T	C	0.0139	0.002	6.20E-12	7.37E-05	48.3	-0.0056	0.0430	8.97E-01
rs2875762	C	G	0.0139	0.002	1.20E-11	7.19E-05	48.3	-0.0131	0.0448	7.71E-01
rs11736228	A	T	0.0139	0.002	4.10E-12	7.41E-05	48.3	0.0197	0.0429	6.49E-01
rs12035149	G	C	0.0146	0.0021	3.90E-12	7.39E-05	48.3	0.0018	0.0448	9.67E-01
rs10510999	T	C	0.0146	0.0021	7.80E-12	6.68E-05	48.3	0.0261	0.0459	5.71E-01
rs6419734	T	C	0.0174	0.0025	1.90E-12	7.81E-05	48.4	0.0188	0.0523	7.20E-01
rs3887080	A	G	0.0181	0.0026	5.10E-12	6.99E-05	48.5	-0.0470	0.0571	4.13E-01
rs12325419	G	A	0.0188	0.0027	1.40E-12	7.52E-05	48.5	0.0147	0.0561	7.94E-01
rs16907751	C	T	0.0209	0.003	1.60E-12	8.19E-05	48.5	0.0394	0.0638	5.39E-01
rs4740383	A	G	0.0126	0.0018	1.90E-12	7.71E-05	49.0	-0.0351	0.0376	3.53E-01
rs17531363	A	C	0.0133	0.0019	2.00E-12	7.48E-05	49.0	-0.0647	0.0408	1.15E-01
rs845084	A	G	0.014	0.002	1.30E-12	7.69E-05	49.0	-0.0301	0.0425	4.81E-01
rs6690764	G	A	0.0154	0.0022	6.30E-12	7.82E-05	49.0	0.0295	0.0453	5.18E-01
rs1512914	G	T	0.0126	0.0018	8.10E-13	7.55E-05	49.0	-0.0257	0.0379	4.99E-01
rs7607351	T	C	0.0119	0.0017	8.40E-12	6.90E-05	49.0	-0.0191	0.0375	6.12E-01
rs16932761	G	A	0.014	0.002	1.70E-12	7.43E-05	49.0	-0.0084	0.0433	8.47E-01
rs2029331	G	C	0.014	0.002	2.00E-12	7.25E-05	49.0	0.0727	0.0440	1.00E-01
rs1814170	A	T	0.0203	0.0029	2.10E-12	7.77E-05	49.0	-0.0472	0.0590	4.26E-01
rs11614340	C	T	0.0133	0.0019	8.50E-13	7.56E-05	49.0	-0.0618	0.0391	1.17E-01
rs10433609	A	T	0.0162	0.0023	1.70E-12	7.35E-05	49.6	-0.0481	0.0486	3.25E-01
rs6443750	C	T	0.0148	0.0021	3.20E-12	6.83E-05	49.7	0.0002	0.0480	9.97E-01
rs7186893	G	T	0.0141	0.002	6.50E-13	7.81E-05	49.7	0.0779	0.0420	6.54E-02
rs4718966	T	C	0.0127	0.0018	4.60E-13	7.85E-05	49.8	-0.0047	0.0379	9.02E-01
rs6449532	C	T	0.0127	0.0018	1.20E-12	7.38E-05	49.8	-0.0028	0.0391	9.44E-01
rs1263618	C	T	0.012	0.0017	5.30E-12	6.21E-05	49.8	-0.0413	0.0399	3.04E-01
rs11066188	G	A	0.012	0.0017	8.10E-13	7.01E-05	49.8	-0.2250	0.0377	2.94E-09
rs1007934	G	A	0.012	0.0017	7.60E-12	6.99E-05	49.8	0.0078	0.0379	8.38E-01
rs4985155	A	G	0.012	0.0017	3.00E-12	6.43E-05	49.8	-0.0286	0.0398	4.74E-01
rs3766430	C	T	0.0113	0.0016	3.90E-12	6.30E-05	49.9	0.0246	0.0376	5.15E-01
rs16966801	G	A	0.0156	0.0022	1.10E-12	7.83E-05	50.3	0.0370	0.0476	4.39E-01
rs6512302	C	G	0.0142	0.002	2.10E-12	7.54E-05	50.4	-0.0105	0.0424	8.06E-01
rs7421089	T	C	0.0135	0.0019	7.50E-13	7.49E-05	50.5	0.0386	0.0408	3.47E-01
rs6504165	T	C	0.0135	0.0019	4.00E-13	7.06E-05	50.5	-0.0174	0.0426	6.84E-01
rs10971721	C	T	0.0199	0.0028	5.70E-13	7.69E-05	50.5	0.0885	0.0553	1.11E-01
rs1707322	G	A	0.0128	0.0018	4.90E-13	6.80E-05	50.6	-0.0671	0.0405	9.97E-02
rs9530843	A	C	0.0128	0.0018	4.80E-13	8.09E-05	50.6	0.0190	0.0375	6.14E-01
rs3811514	T	C	0.0121	0.0017	1.20E-12	7.32E-05	50.7	0.0431	0.0374	2.52E-01
rs765875	C	T	0.0121	0.0017	3.00E-12	7.31E-05	50.7	-0.0025	0.0374	9.48E-01
rs2174307	C	G	0.0121	0.0017	4.90E-12	7.07E-05	50.7	-0.0073	0.0377	8.47E-01
rs1521527	G	C	0.0121	0.0017	3.10E-12	7.29E-05	50.7	-0.0265	0.0374	4.82E-01
rs6595205	C	G	0.0114	0.0016	2.00E-12	6.47E-05	50.8	0.0449	0.0373	2.32E-01
rs4677812	C	A	0.0136	0.0019	1.40E-12	7.47E-05	51.2	-0.0330	0.0414	4.28E-01
rs326889	C	T	0.0129	0.0018	2.40E-13	7.94E-05	51.4	0.0628	0.0378	9.89E-02
rs9571687	C	A	0.0129	0.0018	2.80E-12	7.35E-05	51.4	0.0302	0.0398	4.50E-01
rs4936175	C	T	0.0122	0.0017	1.40E-12	7.35E-05	51.5	-0.0554	0.0379	1.47E-01

SNP	Effect allele	Other allele	SNP-BMI association					SNP-LADA association		
			BETA	SE	*P*	R^2	F statistics	BETA	SE	*P*
rs7313220	A	G	0.0122	0.0017	1.40E-12	7.44E-05	51.5	0.0351	0.0372	3.48E-01
rs9349239	A	G	0.0122	0.0017	1.30E-12	7.44E-05	51.5	-0.0096	0.0370	7.97E-01
rs1955540	C	T	0.0158	0.0022	6.20E-13	7.75E-05	51.6	-0.0108	0.0471	8.20E-01
rs2246012	C	T	0.0158	0.0022	3.10E-13	6.80E-05	51.6	0.0625	0.0504	2.18E-01
rs3845802	G	T	0.0115	0.0016	1.20E-12	6.61E-05	51.7	0.0008	0.0372	9.82E-01
rs4722672	C	T	0.0151	0.0021	1.80E-12	6.87E-05	51.7	0.0284	0.0469	5.47E-01
rs11792311	G	A	0.0144	0.002	1.30E-12	7.54E-05	51.8	0.0070	0.0432	8.72E-01
rs538579	C	G	0.0137	0.0019	1.30E-13	8.21E-05	52.0	0.0525	0.0406	1.99E-01
rs1365466	C	T	0.0137	0.0019	3.30E-13	7.21E-05	52.0	-0.0287	0.0430	5.07E-01
rs12989476	T	C	0.013	0.0018	5.00E-13	7.71E-05	52.2	-0.0198	0.0392	6.15E-01
rs1625427	T	C	0.013	0.0018	1.40E-12	7.69E-05	52.2	-0.0471	0.0393	2.34E-01
rs954018	G	A	0.013	0.0018	2.10E-13	7.17E-05	52.2	-0.0815	0.0402	4.38E-02
rs4963120	T	C	0.013	0.0018	3.60E-13	8.31E-05	52.2	-0.0017	0.0378	9.64E-01
rs945211	C	G	0.013	0.0018	4.50E-13	7.82E-05	52.2	0.0345	0.0392	3.82E-01
rs4148155	A	G	0.0188	0.0026	5.00E-13	7.07E-05	52.3	0.0591	0.0604	3.30E-01
rs2143253	G	A	0.0188	0.0026	1.10E-12	7.41E-05	52.3	0.0564	0.0606	3.54E-01
rs11951673	C	T	0.0123	0.0017	1.10E-13	7.23E-05	52.3	-0.0072	0.0383	8.51E-01
rs1941697	A	G	0.0123	0.0017	1.20E-12	7.50E-05	52.3	0.0226	0.0370	5.43E-01
rs6477694	C	T	0.0123	0.0017	3.80E-13	6.94E-05	52.3	-0.0402	0.0383	2.98E-01
rs2124499	G	C	0.0123	0.0017	3.40E-13	7.07E-05	52.3	-0.0098	0.0382	7.99E-01
rs2467210	G	A	0.0145	0.002	7.90E-13	7.38E-05	52.6	-0.0604	0.0433	1.65E-01
rs4969387	G	C	0.0145	0.002	4.30E-13	7.96E-05	52.6	-0.0294	0.0427	4.93E-01
rs11855853	C	T	0.0145	0.002	2.40E-13	8.19E-05	52.6	0.0074	0.0421	8.61E-01
rs740157	A	G	0.0116	0.0016	1.80E-12	6.60E-05	52.6	-0.0182	0.0383	6.36E-01
rs6448587	A	C	0.0167	0.0023	2.30E-13	8.55E-05	52.7	0.0583	0.0471	2.18E-01
rs9077	G	A	0.0138	0.0019	3.50E-13	8.42E-05	52.8	0.0051	0.0393	8.98E-01
rs2235564	T	C	0.0131	0.0018	3.70E-13	7.77E-05	53.0	0.0612	0.0393	1.22E-01
rs3852012	G	A	0.0131	0.0018	1.40E-13	7.37E-05	53.0	0.0220	0.0398	5.82E-01
rs7239114	A	G	0.0124	0.0017	1.20E-13	7.64E-05	53.2	-0.0012	0.0375	9.74E-01
rs340025	C	T	0.0124	0.0017	1.00E-13	7.53E-05	53.2	0.0080	0.0381	8.35E-01
rs10797115	T	C	0.0124	0.0017	9.90E-13	7.65E-05	53.2	0.0304	0.0373	4.18E-01
rs11629783	C	G	0.0146	0.002	9.80E-13	7.57E-05	53.3	-0.0722	0.0443	1.05E-01
rs12041258	T	C	0.0146	0.002	9.50E-13	7.52E-05	53.3	0.0585	0.0437	1.83E-01
rs7630302	G	C	0.0212	0.0029	5.60E-13	8.07E-05	53.4	0.0927	0.0637	1.48E-01
rs1899689	T	C	0.0117	0.0016	1.50E-12	6.56E-05	53.5	-0.0226	0.0379	5.53E-01
rs7607369	A	G	0.0117	0.0016	9.30E-13	6.74E-05	53.5	-0.0087	0.0380	8.19E-01
rs1038088	G	T	0.0117	0.0016	4.60E-13	6.84E-05	53.5	-0.0281	0.0371	4.52E-01
rs1150659	G	A	0.0139	0.0019	3.80E-13	6.75E-05	53.5	-0.0007	0.0455	9.88E-01
rs10795422	G	A	0.0139	0.0019	9.30E-14	8.26E-05	53.5	0.0533	0.0405	1.91E-01
rs2682406	T	A	0.0132	0.0018	8.90E-14	8.47E-05	53.8	-0.0388	0.0374	3.03E-01
rs1784460	A	T	0.0132	0.0018	9.00E-14	8.39E-05	53.8	0.1013	0.0382	8.44E-03
rs1330052	G	C	0.0132	0.0018	1.50E-13	7.93E-05	53.8	0.0204	0.0391	6.05E-01
rs6138482	T	C	0.0147	0.002	5.80E-13	6.85E-05	54.0	-0.0269	0.0456	5.57E-01
rs16871902	A	G	0.0125	0.0017	4.60E-13	7.81E-05	54.1	-0.0180	0.0374	6.33E-01
rs10832778	G	C	0.0125	0.0017	1.30E-13	7.35E-05	54.1	0.0230	0.0379	5.46E-01
rs329122	G	A	0.0125	0.0017	4.30E-14	7.63E-05	54.1	-0.0147	0.0374	6.96E-01
rs7519259	A	G	0.0125	0.0017	3.80E-13	7.77E-05	54.1	-0.0162	0.0370	6.63E-01
rs6864049	G	A	0.0125	0.0017	6.70E-14	7.76E-05	54.1	0.0175	0.0374	6.42E-01

SNP	Effect allele	Other allele	SNP-BMI association					SNP-LADA association		
			BETA	SE	*P*	R^2	F statistics	BETA	SE	*P*
rs1358980	C	T	0.0125	0.0017	1.10E-13	7.80E-05	54.1	0.0163	0.0376	6.67E-01
rs12439798	T	G	0.0125	0.0017	6.90E-13	7.63E-05	54.1	0.0344	0.0379	3.67E-01
rs977540	A	G	0.014	0.0019	2.50E-13	7.12E-05	54.3	-0.0217	0.0433	6.18E-01
rs7869771	A	C	0.014	0.0019	4.90E-13	7.63E-05	54.3	0.0252	0.0443	5.72E-01
rs10838465	A	C	0.014	0.0019	9.10E-14	8.28E-05	54.3	0.0623	0.0403	1.24E-01
rs12147845	T	C	0.0199	0.0027	4.50E-13	7.97E-05	54.3	-0.0623	0.0590	2.94E-01
rs10923724	C	T	0.0118	0.0016	6.40E-13	6.82E-05	54.4	-0.0047	0.0376	9.02E-01
rs1689437	G	A	0.0251	0.0034	2.10E-13	7.82E-05	54.5	0.0059	0.0815	9.43E-01
rs10007906	A	C	0.0133	0.0018	1.70E-13	8.15E-05	54.6	-0.0056	0.0387	8.86E-01
rs6738445	C	T	0.0133	0.0018	1.90E-13	7.19E-05	54.6	-0.0220	0.0414	5.98E-01
rs11150911	A	C	0.0133	0.0018	4.70E-13	7.15E-05	54.6	0.0260	0.0403	5.21E-01
rs12680842	A	G	0.0133	0.0018	4.40E-14	7.70E-05	54.6	-0.0242	0.0395	5.43E-01
rs2890652	C	T	0.017	0.0023	2.50E-13	8.38E-05	54.6	0.0963	0.0479	4.57E-02
rs9267677	C	T	0.0207	0.0028	1.60E-13	7.79E-05	54.7	-0.0876	0.0722	2.28E-01
rs11945861	G	A	0.0148	0.002	5.00E-13	7.92E-05	54.8	-0.0243	0.0450	5.91E-01
rs10818810	A	G	0.0126	0.0017	1.10E-13	7.56E-05	54.9	0.0327	0.0381	3.93E-01
rs10878946	C	T	0.0141	0.0019	3.60E-13	8.12E-05	55.1	0.1049	0.0408	1.07E-02
rs7235205	G	A	0.0141	0.0019	4.40E-14	7.76E-05	55.1	-0.0182	0.0411	6.60E-01
rs2907948	G	A	0.0141	0.0019	1.30E-13	7.31E-05	55.1	-0.0343	0.0444	4.43E-01
rs2190788	T	G	0.0141	0.0019	2.90E-14	8.51E-05	55.1	-0.0218	0.0403	5.91E-01
rs6471941	A	G	0.0156	0.0021	3.10E-13	6.82E-05	55.2	-0.0316	0.0486	5.17E-01
rs2367112	T	G	0.0119	0.0016	2.30E-13	7.08E-05	55.3	0.0228	0.0373	5.43E-01
rs506338	C	T	0.0134	0.0018	7.80E-14	7.52E-05	55.4	0.0060	0.0406	8.83E-01
rs6445538	C	T	0.0149	0.002	1.40E-13	7.99E-05	55.5	0.0634	0.0433	1.45E-01
rs1522569	T	G	0.0164	0.0022	2.90E-13	8.00E-05	55.6	-0.0032	0.0497	9.48E-01
rs6710871	A	G	0.0179	0.0024	1.00E-13	7.80E-05	55.6	-0.0214	0.0560	7.04E-01
rs3807566	G	T	0.0127	0.0017	2.00E-13	7.96E-05	55.8	-0.0262	0.0376	4.88E-01
rs7874154	C	T	0.0127	0.0017	1.80E-13	8.06E-05	55.8	0.0598	0.0373	1.11E-01
rs559231	T	G	0.0135	0.0018	2.40E-14	8.72E-05	56.3	0.0281	0.0380	4.62E-01
rs769674	A	T	0.0135	0.0018	2.20E-13	7.96E-05	56.3	0.0152	0.0397	7.03E-01
rs7947143	G	A	0.018	0.0024	2.40E-14	8.82E-05	56.3	0.0278	0.0531	6.03E-01
rs2185027	C	A	0.0135	0.0018	5.00E-14	7.61E-05	56.3	0.0261	0.0404	5.21E-01
rs12762034	C	T	0.024	0.0032	7.30E-14	8.07E-05	56.3	0.1264	0.0688	6.77E-02
rs17203016	G	A	0.015	0.002	2.10E-13	7.09E-05	56.3	0.0017	0.0458	9.71E-01
rs2289379	C	T	0.0135	0.0018	4.30E-14	8.73E-05	56.3	-0.0530	0.0380	1.65E-01
rs6707445	A	G	0.0128	0.0017	2.10E-13	8.12E-05	56.7	-0.0586	0.0376	1.21E-01
rs10887578	C	G	0.0128	0.0017	1.60E-13	8.19E-05	56.7	0.0548	0.0373	1.44E-01
rs349088	C	A	0.0128	0.0017	1.80E-13	8.19E-05	56.7	-0.0333	0.0375	3.76E-01
rs12868881	A	T	0.0128	0.0017	1.70E-13	7.98E-05	56.7	0.0395	0.0376	2.96E-01
rs12564992	G	A	0.0196	0.0026	5.30E-14	7.78E-05	56.8	-0.0639	0.0595	2.85E-01
rs10192119	G	T	0.0166	0.0022	3.00E-14	7.68E-05	56.9	-0.0355	0.0493	4.74E-01
rs6548221	A	G	0.0151	0.002	1.70E-14	7.94E-05	57.0	0.0195	0.0460	6.73E-01
rs6548834	A	G	0.0136	0.0018	1.80E-14	8.55E-05	57.1	0.0332	0.0386	3.93E-01
rs7640424	C	T	0.0136	0.0018	2.30E-14	7.72E-05	57.1	0.0253	0.0408	5.37E-01
rs6606686	G	C	0.0136	0.0018	7.60E-15	7.91E-05	57.1	-0.0203	0.0407	6.21E-01
rs1075901	C	T	0.0121	0.0016	1.20E-13	7.20E-05	57.2	0.0161	0.0374	6.68E-01
rs2516739	G	A	0.0159	0.0021	1.40E-14	8.59E-05	57.3	0.0210	0.0469	6.56E-01
rs7561278	T	C	0.0159	0.0021	5.70E-14	8.50E-05	57.3	0.0014	0.0465	9.76E-01

SNP	Effect allele	Other allele	SNP-BMI association					SNP-LADA association		
			BETA	SE	*P*	R^2	F statistics	BETA	SE	*P*
rs6461115	A	G	0.0144	0.0019	1.20E-13	7.31E-05	57.4	-0.0441	0.0433	3.12E-01
rs217671	G	A	0.0144	0.0019	1.30E-13	8.21E-05	57.4	0.0194	0.0423	6.48E-01
rs2192158	A	G	0.0129	0.0017	8.30E-14	8.27E-05	57.6	0.0068	0.0372	8.56E-01
rs2080454	C	A	0.0129	0.0017	1.70E-14	7.83E-05	57.6	-0.0324	0.0381	3.98E-01
rs1554790	G	C	0.0129	0.0017	6.80E-14	8.31E-05	57.6	0.0070	0.0377	8.53E-01
rs10497810	C	T	0.0167	0.0022	5.90E-14	7.98E-05	57.6	0.0779	0.0500	1.21E-01
rs16867703	G	T	0.0137	0.0018	1.40E-14	8.71E-05	57.9	-0.0008	0.0390	9.84E-01
rs11264483	C	G	0.0137	0.0018	3.00E-14	8.91E-05	57.9	0.0241	0.0382	5.32E-01
rs1158805	C	A	0.0137	0.0018	1.20E-14	8.81E-05	57.9	0.0020	0.0380	9.58E-01
rs2246664	A	G	0.0137	0.0018	1.80E-14	8.71E-05	57.9	0.0129	0.0389	7.42E-01
rs1951455	C	T	0.0145	0.0019	4.50E-14	8.38E-05	58.2	0.0093	0.0415	8.24E-01
rs1876359	T	C	0.013	0.0017	1.50E-14	7.79E-05	58.5	-0.0178	0.0395	6.53E-01
rs7217226	G	T	0.013	0.0017	2.60E-14	7.78E-05	58.5	-0.0249	0.0388	5.24E-01
rs733594	T	C	0.0138	0.0018	5.90E-14	7.71E-05	58.8	-0.0073	0.0416	8.61E-01
rs10510419	G	T	0.0177	0.0023	2.20E-14	7.62E-05	59.2	-0.0164	0.0535	7.61E-01
rs9650755	G	A	0.0154	0.002	2.80E-15	9.27E-05	59.3	-0.0187	0.0420	6.59E-01
rs13292976	T	C	0.0131	0.0017	3.10E-14	8.48E-05	59.4	0.0183	0.0373	6.25E-01
rs2163188	C	G	0.0131	0.0017	2.00E-14	8.56E-05	59.4	0.0229	0.0368	5.37E-01
rs10779751	A	G	0.0139	0.0018	2.50E-14	7.69E-05	59.6	0.0305	0.0419	4.69E-01
rs7780752	C	T	0.0139	0.0018	1.00E-14	8.90E-05	59.6	-0.0011	0.0388	9.77E-01
rs284227	C	T	0.0147	0.0019	3.50E-15	8.21E-05	59.9	0.0420	0.0424	3.25E-01
rs6019482	C	T	0.0178	0.0023	2.80E-14	8.75E-05	59.9	-0.0250	0.0484	6.07E-01
rs3849570	A	C	0.0132	0.0017	3.30E-14	7.84E-05	60.3	-0.0755	0.0386	5.15E-02
rs12705977	T	G	0.0132	0.0017	2.10E-14	8.64E-05	60.3	0.0321	0.0371	3.89E-01
rs998732	A	G	0.0171	0.0022	2.00E-14	7.77E-05	60.4	-0.0146	0.0519	7.80E-01
rs13012099	G	A	0.014	0.0018	1.20E-14	8.80E-05	60.5	-0.0164	0.0391	6.77E-01
rs1045411	C	T	0.0148	0.0019	2.30E-15	8.53E-05	60.7	0.0980	0.0421	2.06E-02
rs7871866	C	G	0.0187	0.0024	2.30E-14	9.07E-05	60.7	-0.0196	0.0534	7.15E-01
rs7536433	C	T	0.0156	0.002	9.80E-15	7.96E-05	60.8	-0.0626	0.0454	1.70E-01
rs329651	T	G	0.0164	0.0021	9.00E-15	8.43E-05	61.0	-0.0427	0.0493	3.90E-01
rs17327461	T	C	0.0125	0.0016	1.50E-14	7.73E-05	61.0	0.0094	0.0373	8.03E-01
rs10269783	A	G	0.0133	0.0017	1.40E-15	8.41E-05	61.2	0.0018	0.0378	9.63E-01
rs7195386	T	C	0.0133	0.0017	1.10E-14	8.84E-05	61.2	-0.0154	0.0374	6.83E-01
rs4012234	G	T	0.0141	0.0018	9.90E-16	9.60E-05	61.4	0.0108	0.0379	7.78E-01
rs1452075	T	C	0.0141	0.0018	1.30E-14	7.88E-05	61.4	-0.0243	0.0422	5.66E-01
rs13290794	G	A	0.0141	0.0018	1.90E-15	9.14E-05	61.4	0.0582	0.0382	1.30E-01
rs1336486	G	T	0.0141	0.0018	1.80E-14	8.66E-05	61.4	-0.0876	0.0404	3.11E-02
rs7601895	C	G	0.0149	0.0019	1.70E-15	9.25E-05	61.5	-0.0265	0.0417	5.28E-01
rs905938	C	T	0.0149	0.0019	1.20E-15	8.70E-05	61.5	-0.0132	0.0414	7.52E-01
rs12299814	C	A	0.0157	0.002	5.20E-15	9.30E-05	61.6	-0.0087	0.0419	8.37E-01
rs155510	T	G	0.0165	0.0021	1.70E-14	8.42E-05	61.7	-0.0239	0.0457	6.03E-01
rs2282231	T	C	0.0165	0.0021	4.80E-15	9.49E-05	61.7	0.0491	0.0441	2.68E-01
rs11889536	A	G	0.0189	0.0024	6.40E-15	9.07E-05	62.0	0.0241	0.0547	6.61E-01
rs10460960	A	G	0.0197	0.0025	8.10E-15	7.74E-05	62.1	0.1239	0.0562	2.85E-02
rs4372836	T	C	0.0142	0.0018	7.30E-16	8.52E-05	62.2	-0.0101	0.0409	8.06E-01
rs7573263	T	C	0.0142	0.0018	1.60E-15	9.75E-05	62.2	0.0232	0.0376	5.40E-01
rs33485	C	T	0.0158	0.002	1.10E-15	9.82E-05	62.4	-0.0366	0.0415	3.80E-01
rs946824	T	C	0.0206	0.0026	1.10E-15	1.03E-04	62.8	-0.0342	0.0574	5.53E-01

SNP	Effect allele	Other allele	SNP-BMI association					SNP-LADA association		
			BETA	SE	*P*	R^2	F statistics	BETA	SE	*P*
rs8088123	C	A	0.0262	0.0033	3.70E-15	9.75E-05	63.0	-0.0159	0.0723	8.27E-01
rs17820822	T	G	0.0143	0.0018	2.90E-15	9.37E-05	63.1	-0.0008	0.0383	9.84E-01
rs3209570	G	A	0.0143	0.0018	9.80E-16	9.58E-05	63.1	0.0930	0.0386	1.68E-02
rs4711986	A	G	0.0143	0.0018	1.70E-15	9.35E-05	63.1	-0.0167	0.0391	6.71E-01
rs8097672	T	A	0.02	0.0025	8.40E-16	1.04E-04	64.0	-0.0314	0.0519	5.48E-01
rs2281819	T	A	0.016	0.002	5.10E-15	9.07E-05	64.0	0.0942	0.0444	3.49E-02
rs3957285	A	G	0.0144	0.0018	2.90E-16	1.03E-04	64.0	0.0207	0.0380	5.89E-01
rs10939792	G	C	0.0152	0.0019	1.40E-15	1.02E-04	64.0	0.0247	0.0396	5.36E-01
rs12454712	C	T	0.0144	0.0018	4.40E-16	9.67E-05	64.0	-0.0556	0.0387	1.53E-01
rs10886017	A	C	0.0152	0.0019	1.40E-15	8.72E-05	64.0	-0.0518	0.0436	2.38E-01
rs13380104	C	T	0.0136	0.0017	7.70E-15	9.00E-05	64.0	-0.0031	0.0379	9.35E-01
rs2271189	G	A	0.0144	0.0018	5.00E-16	9.99E-05	64.0	-0.1384	0.0378	2.75E-04
rs9827823	T	C	0.0193	0.0024	1.20E-15	9.44E-05	64.7	0.0289	0.0532	5.89E-01
rs10146527	T	C	0.0137	0.0017	2.30E-15	8.69E-05	64.9	-0.0094	0.0390	8.10E-01
rs10842240	C	G	0.0218	0.0027	3.10E-16	9.73E-05	65.2	0.0454	0.0594	4.47E-01
rs498240	G	A	0.0267	0.0033	1.60E-15	8.74E-05	65.5	0.3216	0.0800	6.52E-05
rs12364470	G	T	0.0178	0.0022	1.10E-15	8.63E-05	65.5	0.0544	0.0496	2.76E-01
rs12593036	A	G	0.0154	0.0019	3.80E-16	9.95E-05	65.7	0.0666	0.0401	9.88E-02
rs12033257	A	G	0.0146	0.0018	2.40E-15	1.01E-04	65.8	0.0178	0.0382	6.43E-01
rs1895957	G	T	0.0171	0.0021	1.40E-16	1.01E-04	66.3	-0.0897	0.0447	4.61E-02
rs7925214	T	C	0.0147	0.0018	4.40E-17	1.08E-04	66.7	0.0381	0.0372	3.09E-01
rs8123881	G	A	0.0196	0.0024	4.40E-16	8.68E-05	66.7	0.1338	0.0522	1.08E-02
rs2228213	G	A	0.0139	0.0017	4.60E-16	8.77E-05	66.9	0.0544	0.0383	1.58E-01
rs9540493	A	G	0.0139	0.0017	8.10E-17	9.53E-05	66.9	0.0031	0.0375	9.33E-01
rs6841761	G	T	0.0131	0.0016	6.40E-16	8.56E-05	67.0	-0.0451	0.0373	2.30E-01
rs4556997	A	C	0.0197	0.0024	6.90E-17	9.06E-05	67.4	-0.0255	0.0556	6.48E-01
rs10942267	A	G	0.0156	0.0019	3.90E-17	1.04E-04	67.4	-0.0003	0.0404	9.94E-01
rs11866815	C	T	0.0156	0.0019	1.00E-16	9.03E-05	67.4	0.0259	0.0445	5.63E-01
rs7730004	T	C	0.0148	0.0018	9.10E-16	9.70E-05	67.6	-0.0381	0.0393	3.35E-01
rs536445	T	C	0.014	0.0017	4.60E-16	9.77E-05	67.8	-0.0224	0.0374	5.52E-01
rs1503526	C	T	0.014	0.0017	5.50E-17	9.79E-05	67.8	-0.0667	0.0375	7.70E-02
rs1436344	C	G	0.0141	0.0017	4.10E-16	9.60E-05	68.8	-0.0080	0.0379	8.33E-01
rs811054	T	C	0.0141	0.0017	2.70E-17	9.89E-05	68.8	-0.0437	0.0378	2.50E-01
rs4864201	T	C	0.0141	0.0017	1.50E-16	9.08E-05	68.8	0.0330	0.0389	4.00E-01
rs13184896	G	T	0.0133	0.0016	3.30E-16	8.69E-05	69.1	-0.0146	0.0376	6.98E-01
rs13174863	G	A	0.0192	0.0023	2.90E-16	9.65E-05	69.7	-0.0461	0.0509	3.68E-01
rs6692586	A	G	0.0192	0.0023	1.10E-16	1.03E-04	69.7	0.0154	0.0502	7.60E-01
rs10145461	G	T	0.0142	0.0017	4.70E-16	1.00E-04	69.8	0.0041	0.0371	9.12E-01
rs3007105	T	C	0.0142	0.0017	1.10E-17	1.00E-04	69.8	0.0291	0.0378	4.44E-01
rs4673553	G	T	0.0142	0.0017	2.10E-16	1.00E-04	69.8	0.0173	0.0376	6.47E-01
rs3736485	A	G	0.0134	0.0016	2.50E-16	8.91E-05	70.1	-0.0495	0.0380	1.95E-01
rs12602912	T	C	0.0176	0.0021	9.90E-18	1.01E-04	70.2	-0.0701	0.0463	1.32E-01
rs17636031	C	T	0.016	0.0019	1.20E-17	1.01E-04	70.9	0.0457	0.0432	2.93E-01
rs29941	G	A	0.0152	0.0018	5.10E-18	9.94E-05	71.3	-0.0052	0.0397	8.96E-01
rs13209872	G	C	0.0152	0.0018	1.40E-16	1.04E-04	71.3	0.0749	0.0396	6.01E-02
rs215634	A	G	0.0152	0.0018	2.60E-17	1.09E-04	71.3	-0.0050	0.0386	8.97E-01
rs8192675	C	T	0.0152	0.0018	1.40E-17	9.49E-05	71.3	0.1046	0.0403	9.83E-03
rs7535528	G	A	0.0152	0.0018	1.40E-16	1.08E-04	71.3	-0.0174	0.0385	6.53E-01

SNP	Effect allele	Other allele	SNP-BMI association					SNP-LADA association		
			BETA	SE	*P*	R^2	F statistics	BETA	SE	*P*
rs995258	A	C	0.0144	0.0017	4.50E-17	1.01E-04	71.8	-0.0799	0.0376	3.45E-02
rs13417156	C	T	0.0144	0.0017	2.60E-17	1.02E-04	71.8	0.0007	0.0375	9.84E-01
rs273504	G	A	0.0153	0.0018	4.40E-18	1.15E-04	72.3	0.0365	0.0379	3.38E-01
rs13240600	A	G	0.0204	0.0024	3.50E-17	1.09E-04	72.3	-0.0753	0.0512	1.44E-01
rs2357760	A	G	0.0145	0.0017	6.80E-17	9.22E-05	72.8	0.0039	0.0395	9.22E-01
rs12477088	T	C	0.0145	0.0017	8.90E-17	1.01E-04	72.8	-0.0111	0.0381	7.72E-01
rs13047416	C	G	0.0154	0.0018	2.20E-17	1.11E-04	73.2	0.0046	0.0391	9.06E-01
rs2479958	A	G	0.0154	0.0018	1.50E-17	1.19E-04	73.2	0.0063	0.0373	8.67E-01
rs13263601	C	A	0.0154	0.0018	2.20E-17	1.08E-04	73.2	0.0002	0.0398	9.97E-01
rs1000940	G	A	0.0154	0.0018	1.10E-17	9.94E-05	73.2	0.0119	0.0409	7.73E-01
rs901630	C	T	0.0146	0.0017	1.90E-18	1.02E-04	73.8	0.0077	0.0380	8.41E-01
rs12454204	T	G	0.0172	0.002	3.80E-17	1.05E-04	74.0	-0.1048	0.0435	1.67E-02
rs10962549	T	C	0.0198	0.0023	2.50E-17	1.10E-04	74.1	0.0694	0.0513	1.79E-01
rs2162524	C	T	0.0155	0.0018	4.10E-17	1.07E-04	74.2	0.0517	0.0393	1.91E-01
rs592483	C	T	0.0147	0.0017	2.00E-18	1.06E-04	74.8	-0.0127	0.0371	7.34E-01
rs6587552	A	G	0.0173	0.002	1.60E-17	1.09E-04	74.8	0.0291	0.0432	5.02E-01
rs1006896	A	C	0.0234	0.0027	5.50E-18	1.04E-04	75.1	-0.0470	0.0605	4.40E-01
rs4500930	T	C	0.0156	0.0018	6.50E-18	1.10E-04	75.1	0.0520	0.0386	1.81E-01
rs1852006	G	A	0.0156	0.0018	4.90E-18	1.11E-04	75.1	-0.0030	0.0391	9.39E-01
rs9507983	C	T	0.0156	0.0018	1.40E-18	1.16E-04	75.1	-0.0171	0.0382	6.57E-01
rs756717	G	A	0.0148	0.0017	5.40E-18	1.05E-04	75.8	-0.0026	0.0382	9.46E-01
rs7025938	G	C	0.0166	0.0019	3.70E-19	1.20E-04	76.3	-0.0589	0.0399	1.42E-01
rs10197031	C	T	0.0166	0.0019	1.90E-18	1.12E-04	76.3	0.0630	0.0408	1.25E-01
rs895330	C	G	0.0201	0.0023	5.50E-19	1.26E-04	76.4	-0.0325	0.0464	4.85E-01
rs11084553	A	G	0.021	0.0024	1.80E-18	1.14E-04	76.6	0.0705	0.0551	2.04E-01
rs7102454	C	T	0.0158	0.0018	2.40E-18	1.13E-04	77.0	-0.0396	0.0397	3.21E-01
rs1928295	T	C	0.0141	0.0016	5.40E-18	9.82E-05	77.7	0.0086	0.0375	8.19E-01
rs429343	A	G	0.015	0.0017	6.80E-18	1.10E-04	77.9	-0.0144	0.0381	7.06E-01
rs2478879	A	G	0.0159	0.0018	3.80E-19	1.21E-04	78.0	-0.0142	0.0381	7.10E-01
rs4820408	T	G	0.0151	0.0017	2.10E-19	1.10E-04	78.9	-0.0378	0.0382	3.25E-01
rs7557796	T	C	0.016	0.0018	2.30E-19	1.16E-04	79.0	-0.0058	0.0395	8.85E-01
rs11191548	C	T	0.0268	0.003	5.00E-19	1.08E-04	79.8	0.1048	0.0618	9.19E-02
rs10929925	C	A	0.0143	0.0016	1.80E-18	1.00E-04	79.9	-0.0338	0.0373	3.67E-01
rs12044597	G	A	0.0143	0.0016	1.70E-18	1.02E-04	79.9	-0.0380	0.0373	3.12E-01
rs2396625	T	A	0.0162	0.0018	3.60E-20	1.29E-04	81.0	0.0023	0.0378	9.51E-01
rs355777	C	G	0.0153	0.0017	1.40E-18	1.13E-04	81.0	-0.0658	0.0379	8.41E-02
rs17094222	C	T	0.0181	0.002	2.20E-19	1.06E-04	81.9	-0.0326	0.0468	4.89E-01
rs4639527	G	A	0.0172	0.0019	3.30E-20	1.25E-04	82.0	0.0591	0.0405	1.46E-01
rs7181498	T	C	0.0163	0.0018	1.00E-19	1.24E-04	82.0	0.0022	0.0387	9.54E-01
rs930295	A	C	0.0211	0.0023	1.00E-19	1.19E-04	84.2	0.0234	0.0495	6.39E-01
rs6804842	G	A	0.0156	0.0017	3.60E-21	1.19E-04	84.2	-0.0028	0.0378	9.40E-01
rs9294260	A	G	0.0147	0.0016	1.80E-19	1.08E-04	84.4	0.0352	0.0374	3.48E-01
rs12448257	A	G	0.0184	0.002	8.10E-20	1.15E-04	84.6	0.0166	0.0463	7.21E-01
rs6235	G	C	0.0175	0.0019	1.50E-19	1.21E-04	84.8	-0.0942	0.0411	2.28E-02
rs12630999	A	G	0.0175	0.0019	8.50E-21	1.16E-04	84.8	0.0118	0.0435	7.88E-01
rs2065418	T	G	0.0166	0.0018	3.60E-20	1.27E-04	85.0	-0.0126	0.0390	7.48E-01
rs13227658	C	T	0.0157	0.0017	1.70E-19	1.22E-04	85.3	-0.0060	0.0374	8.74E-01
rs2733287	C	A	0.0157	0.0017	6.80E-20	1.23E-04	85.3	0.0014	0.0370	9.70E-01

SNP	Effect allele	Other allele	SNP-BMI association					SNP-LADA association		
			BETA	SE	*P*	R^2	F statistics	BETA	SE	*P*
rs7243357	T	G	0.0194	0.0021	9.10E-20	1.08E-04	85.3	0.0711	0.0504	1.61E-01
rs1927790	C	T	0.0148	0.0016	1.80E-19	1.06E-04	85.6	0.1009	0.0381	8.55E-03
rs7730898	A	G	0.0168	0.0018	4.50E-20	1.12E-04	87.1	-0.0073	0.0413	8.61E-01
rs7332115	T	G	0.0159	0.0017	3.60E-21	1.18E-04	87.5	0.0150	0.0385	6.99E-01
rs11074446	T	C	0.0225	0.0024	1.80E-20	1.15E-04	87.9	0.0200	0.0553	7.19E-01
rs4757144	A	G	0.0169	0.0018	5.60E-22	1.38E-04	88.2	-0.0467	0.0383	2.26E-01
rs2694047	G	A	0.0188	0.002	3.90E-21	1.34E-04	88.4	0.0509	0.0424	2.33E-01
rs1048932	C	A	0.016	0.0017	3.80E-22	1.24E-04	88.6	0.0244	0.0376	5.19E-01
rs13191362	A	G	0.0236	0.0025	5.90E-21	1.17E-04	89.1	0.0285	0.0577	6.23E-01
rs2010281	G	A	0.0161	0.0017	6.70E-21	1.19E-04	89.7	0.0205	0.0386	5.99E-01
rs1477199	G	A	0.0228	0.0024	9.40E-22	1.29E-04	90.3	0.0579	0.0526	2.74E-01
rs4483850	A	T	0.0162	0.0017	5.20E-21	1.31E-04	90.8	0.0025	0.0374	9.47E-01
rs9989141	T	C	0.0162	0.0017	3.60E-21	1.21E-04	90.8	0.1113	0.0388	4.31E-03
rs12150665	T	C	0.0162	0.0017	1.60E-22	1.27E-04	90.8	-0.0259	0.0374	4.91E-01
rs12468863	C	T	0.0153	0.0016	5.10E-21	1.17E-04	91.4	-0.0525	0.0375	1.63E-01
rs17724992	A	G	0.0183	0.0019	1.00E-22	1.29E-04	92.8	0.0252	0.0434	5.65E-01
rs9538141	A	G	0.0164	0.0017	3.50E-21	1.34E-04	93.1	0.0054	0.0370	8.85E-01
rs1528435	T	C	0.0164	0.0017	9.10E-23	1.25E-04	93.1	0.0547	0.0385	1.57E-01
rs10920678	A	G	0.0155	0.0016	1.50E-21	1.18E-04	93.8	0.0641	0.0374	8.87E-02
rs3800229	T	G	0.0175	0.0018	1.40E-22	1.26E-04	94.5	0.0484	0.0417	2.49E-01
rs577525	C	T	0.0166	0.0017	9.70E-22	1.35E-04	95.3	0.1012	0.0379	8.03E-03
rs1361739	G	A	0.0176	0.0018	6.90E-22	1.36E-04	95.6	0.0348	0.0403	3.90E-01
rs7599312	G	A	0.0186	0.0019	6.90E-24	1.35E-04	95.8	-0.0664	0.0425	1.20E-01
rs1218822	A	G	0.0168	0.0017	1.90E-22	1.26E-04	97.7	-0.0084	0.0396	8.32E-01
rs11079849	C	T	0.0188	0.0019	4.80E-24	1.54E-04	97.9	0.0656	0.0400	1.03E-01
rs11496125	T	C	0.0169	0.0017	3.00E-22	1.39E-04	98.8	0.0147	0.0375	6.96E-01
rs977747	T	G	0.0169	0.0017	1.30E-24	1.38E-04	98.8	0.0347	0.0382	3.67E-01
rs1296328	A	C	0.0179	0.0018	4.90E-24	1.57E-04	98.9	0.0276	0.0374	4.64E-01
rs427943	C	A	0.017	0.0017	7.30E-23	1.42E-04	100.0	-0.0226	0.0376	5.50E-01
rs4482463	C	A	0.0331	0.0033	2.80E-23	1.59E-04	100.6	0.0785	0.0692	2.59E-01
rs3803286	A	G	0.0181	0.0018	4.10E-23	1.48E-04	101.1	0.0340	0.0394	3.91E-01
rs7903146	C	T	0.0181	0.0018	1.30E-23	1.35E-04	101.1	-0.0894	0.0412	3.11E-02
rs2481665	T	C	0.0161	0.0016	7.20E-23	1.28E-04	101.3	-0.0632	0.0375	9.44E-02
rs7084454	A	G	0.0193	0.0019	4.00E-25	1.66E-04	103.2	-0.0764	0.0398	5.66E-02
rs6870983	C	T	0.0204	0.002	6.50E-25	1.42E-04	104.0	0.1054	0.0467	2.50E-02
rs7488867	C	T	0.0204	0.002	8.40E-24	1.62E-04	104.0	0.0265	0.0430	5.40E-01
rs2365389	C	T	0.0174	0.0017	1.30E-25	1.47E-04	104.8	0.0419	0.0378	2.70E-01
rs4986044	C	T	0.0164	0.0016	3.30E-23	1.34E-04	105.1	-0.0115	0.0374	7.60E-01
rs1431659	A	G	0.0196	0.0019	6.00E-24	1.50E-04	106.4	-0.0084	0.0424	8.45E-01
rs4516268	C	A	0.0217	0.0021	5.20E-25	1.46E-04	106.8	0.0789	0.0469	9.46E-02
rs17513613	C	T	0.0186	0.0018	3.60E-26	1.51E-04	106.8	-0.0050	0.0395	8.99E-01
rs7715256	G	T	0.0166	0.0016	2.20E-24	1.34E-04	107.6	0.0080	0.0376	8.33E-01
rs6985109	G	A	0.0177	0.0017	1.50E-26	1.56E-04	108.4	-0.0297	0.0505	5.59E-01
rs208015	T	C	0.0356	0.0034	1.40E-25	1.83E-04	109.6	-0.1078	0.0738	1.47E-01
rs16851483	T	G	0.0369	0.0035	3.20E-26	1.76E-04	111.2	0.0711	0.0755	3.49E-01
rs2075650	A	G	0.0244	0.0023	1.50E-25	1.43E-04	112.5	0.0519	0.0527	3.27E-01
rs4929923	C	T	0.0181	0.0017	7.20E-27	1.51E-04	113.4	0.0083	0.0387	8.32E-01
rs7164727	T	C	0.0182	0.0017	3.30E-25	1.44E-04	114.6	0.0287	0.0402	4.77E-01

SNP	Effect allele	Other allele	SNP-BMI association					SNP-LADA association		
			BETA	SE	*P*	R^2	F statistics	BETA	SE	*P*
rs12885454	C	A	0.0185	0.0017	2.40E-27	1.54E-04	118.4	0.0346	0.0386	3.73E-01
rs657452	A	G	0.0188	0.0017	7.20E-29	1.66E-04	122.3	0.0033	0.0381	9.31E-01
rs11880870	A	G	0.0189	0.0017	1.00E-28	1.78E-04	123.6	0.0398	0.0372	2.89E-01
rs2245368	C	T	0.0257	0.0023	4.90E-28	1.90E-04	124.9	-0.0382	0.0499	4.47E-01
rs1579557	T	C	0.0213	0.0019	1.10E-29	1.90E-04	125.7	0.0713	0.0408	8.21E-02
rs6545714	G	A	0.0191	0.0017	9.10E-31	1.73E-04	126.2	0.0581	0.0382	1.31E-01
rs12939549	A	G	0.018	0.0016	2.70E-28	1.59E-04	126.6	-0.0037	0.0374	9.22E-01
rs1884897	G	A	0.0194	0.0017	1.30E-30	1.75E-04	130.2	0.0091	0.0388	8.16E-01
rs7551507	C	T	0.0184	0.0016	9.30E-30	1.67E-04	132.3	0.0331	0.0378	3.84E-01
rs40067	G	A	0.0266	0.0023	7.10E-30	2.01E-04	133.8	0.0376	0.0501	4.56E-01
rs12369179	C	T	0.0359	0.0031	2.50E-31	2.06E-04	134.1	0.0556	0.0673	4.12E-01
rs4740619	T	C	0.0186	0.0016	2.30E-30	1.71E-04	135.1	0.0232	0.0374	5.38E-01
rs17806379	C	T	0.0258	0.0022	1.50E-30	1.96E-04	137.5	0.0737	0.0496	1.39E-01
rs4237643	T	G	0.0223	0.0019	4.30E-33	2.11E-04	137.8	0.0296	0.0406	4.69E-01
rs2122042	T	G	0.0235	0.002	2.30E-31	1.80E-04	138.1	0.0602	0.0458	1.92E-01
rs1454687	C	G	0.0202	0.0017	5.20E-32	2.04E-04	141.2	-0.0327	0.0373	3.84E-01
rs4889606	A	G	0.0202	0.0017	2.80E-33	1.92E-04	141.2	-0.0390	0.0379	3.06E-01
rs12964689	A	G	0.0203	0.0017	5.10E-32	2.06E-04	142.6	-0.0058	0.0375	8.77E-01
rs17405819	T	C	0.0215	0.0018	4.30E-33	1.95E-04	142.7	0.0234	0.0404	5.65E-01
rs1320903	A	G	0.0216	0.0018	9.20E-32	2.02E-04	144.0	0.0288	0.0402	4.77E-01
rs11611246	T	G	0.024	0.002	5.00E-32	1.91E-04	144.0	-0.0248	0.0458	5.91E-01
rs11165643	T	C	0.0206	0.0017	1.40E-35	2.06E-04	146.8	-0.0225	0.0379	5.55E-01
rs889398	C	T	0.0196	0.0016	1.30E-32	1.88E-04	150.1	0.0656	0.0378	8.42E-02
rs17207196	C	T	0.0221	0.0018	2.10E-35	2.37E-04	150.7	0.0117	0.0383	7.61E-01
rs10132280	C	A	0.0223	0.0018	5.60E-35	2.10E-04	153.5	-0.0008	0.0401	9.85E-01
rs12429545	A	G	0.0316	0.0025	9.60E-38	2.18E-04	159.8	-0.0022	0.0547	9.68E-01
rs17391694	T	C	0.0317	0.0025	7.50E-38	2.11E-04	160.8	0.0876	0.0571	1.28E-01
rs16903285	C	T	0.0331	0.0026	7.60E-38	2.65E-04	162.1	0.0147	0.0560	7.94E-01
rs879620	T	C	0.0231	0.0018	5.30E-38	2.52E-04	164.7	-0.0135	0.0384	7.26E-01
rs4671328	T	G	0.0219	0.0017	2.20E-36	2.37E-04	166.0	0.0420	0.0376	2.66E-01
rs2820311	G	A	0.0235	0.0018	4.10E-38	2.47E-04	170.4	0.0791	0.0389	4.34E-02
rs3814883	T	C	0.0232	0.0017	1.10E-40	2.69E-04	186.2	-0.0455	0.0373	2.26E-01
rs2744974	T	C	0.0249	0.0018	1.40E-45	2.77E-04	191.4	0.0512	0.0399	2.03E-01
rs11713193	A	G	0.0239	0.0017	2.40E-44	2.86E-04	197.7	0.0183	0.0372	6.24E-01
rs7144011	T	G	0.0282	0.002	5.20E-47	2.67E-04	198.8	0.0091	0.0459	8.44E-01
rs1412235	C	G	0.0246	0.0017	6.00E-45	2.62E-04	209.4	-0.0479	0.0402	2.36E-01
rs13329567	C	T	0.0293	0.002	1.00E-50	3.05E-04	214.6	-0.1082	0.0452	1.74E-02
rs12446632	G	A	0.0352	0.0024	2.90E-50	3.03E-04	215.1	-0.0275	0.0544	6.15E-01
rs13107325	T	C	0.047	0.0032	1.10E-47	3.02E-04	215.7	-0.0029	0.0784	9.71E-01
rs3810291	A	G	0.0274	0.0018	2.10E-52	3.32E-04	231.7	-0.0481	0.0399	2.31E-01
rs9816226	T	A	0.0323	0.0021	1.60E-52	3.08E-04	236.6	-0.0158	0.0493	7.50E-01
rs1993709	G	A	0.0331	0.0021	1.90E-57	3.27E-04	248.4	0.0901	0.0477	6.04E-02
rs7498665	G	A	0.0271	0.0017	5.60E-60	3.54E-04	254.1	-0.0165	0.0381	6.67E-01
rs11672660	C	T	0.034	0.0021	1.70E-60	3.77E-04	262.1	-0.0670	0.0449	1.38E-01
rs7124681	A	C	0.0263	0.0016	3.20E-58	3.35E-04	270.2	-0.0202	0.0382	5.99E-01
rs2307111	T	C	0.0265	0.0016	1.60E-58	3.36E-04	274.3	0.0952	0.0382	1.33E-02
rs7138803	A	G	0.03	0.0017	2.30E-71	4.23E-04	311.4	0.0204	0.0380	5.94E-01
rs987237	G	A	0.0409	0.0021	9.30E-84	4.94E-04	379.3	0.0311	0.0477	5.16E-01

SNP	Effect allele	Other allele	SNP-BMI association					SNP-LADA association		
			BETA	SE	*P*	R^2	F statistics	BETA	SE	*P*
rs6265	C	T	0.0412	0.0021	1.00E-86	5.33E-04	384.9	0.0489	0.0470	3.01E-01
rs10938397	G	A	0.0324	0.0016	3.40E-86	5.15E-04	410.1	-0.0167	0.0380	6.61E-01
rs10182181	G	A	0.0325	0.0016	6.70E-90	5.27E-04	412.6	0.0072	0.0373	8.49E-01
rs543874	G	A	0.0475	0.002	1.20E-122	7.09E-04	564.1	-0.0031	0.0455	9.46E-01
rs13021737	G	A	0.0574	0.0021	7.50E-157	9.21E-04	747.1	-0.0171	0.0493	7.30E-01
rs663129	A	G	0.0545	0.0019	1.60E-178	1.05E-03	822.8	0.0856	0.0434	5.00E-02
rs8047395	A	G	0.0642	0.0017	1.00E-305	2.06E-03	1426.2	0.0783	0.0370	3.56E-02

LADA: latent autoimmune diabetes in adults.

ESM Table 4. SNPs excluded from conservative analyses

Exposures	Excluded instruments	Reasons for exclusion
**Birthweight**		
**Conservative analysis 1** [a]	SNPs associated with diabetes-related traits (any type of diabetes, 2 hour fasting glucose, HbA1c, insulin, and so on) at $p<0.001$ (Bonferroni-corrected nominal significance level: 0.05/43).	To minimize the possibility that IVs affect LADA directly (pathway ② in **ESM Fig. 3A**).
**Conservative analysis 2** [a]	SNPs excluded from conservative analysis 1, and SNPs associated with any trait (except body size-related traits at birth) at $p<5\times10^{-8}$.	To further minimize the possibility of pleiotropy.
**BMI in adulthood**		
**Conservative analysis 1** [a]	SNPs associated with diabetes-related traits (any type of diabetes, 2 hour fasting glucose, HbA1c, insulin, and so on) at $p<6\times10^{-5}$ (0.05/820) or any trait (except body size-related traits in adulthood) at $p<5\times10^{-8}$.	Excluding SNPs associated with diabetes-related traits is to reduce the possibility that IVs affect LADA directly (pathway ② in **ESM Fig. 3B**).
**Conservative analysis 2** [a]	SNPs excluded from conservative analysis 1, and SNPs associated with smoking, alcohol, or physical activity at $p<6\times10^{-5}$ (0.05/820) [b].	To reduce the possibility that IVs for adult BMI affect LADA through lifestyle factors (pathway ③ in **ESM Fig. 3B**).
**Conservative analysis 3** [a]	SNPs excluded from conservative analysis 2, and SNPs associated with body size-related traits at birth at $p<6\times10^{-5}$ (0.05/820) [c].	To minimize the violation of IV assumption as shown in pathway ③ in **ESM Fig. 3B**.

[a] The association between SNPs and traits were identified from Phenoscanner[4, 5].

[b] The corresponding conservative analysis were not performed for birthweight since there were no further SNPs associated with smoking, alcohol or physical activity at nominal significance level (Bonferroni-corrected: 0.05/43) after the conservative analysis 2 of birthweight.

[c] The corresponding conservative analysis was not performed for birthweight since there will be too few remaining SNPs (only 7) to be analyzed after further excluding SNPs associated with adult BMI at nominal significance level (Bonferroni-corrected: 0.05/43) from the SNPs in conservative analysis 2 of birthweight. Birthweight and adult BMI can be in the same causal pathway as shown in ESM Fig. 3C. Even if they are in different pathways, the potential pleiotropy from adult BMI will lead to the attenuation rather than the overestimation of OR for birthweight since the genome-wide genetic correlation between birthweight and adult BMI is positive[1].

ESM Table 5. Conservative analyses for the association of birthweight and adult BMI with LADA

Exposures	Conservative analyses [a]	No. of excluded SNPs	No. of included SNPs	OR (95% CI) [g]	*p* for risk estimate	*p* for heterogeneity
**Birthweight**	1 [b]	12	31	1.97 (1.01, 3.84)	0.048	0.426
	2 [c]	30	13	1.80 (0.58, 5.57)	0.305	0.821
**BMI in adulthood**	1 [d]	364	456	1.56 (1.16, 2.09)	0.003	0.815
	2 [e]	396	424	1.57 (1.16, 2.14)	0.004	0.909
	3 [f]	399	421	1.59 (1.17, 2.16)	0.003	0.894

LADA latent autoimmune diabetes in adults.

[a] The method of inverse-variance weighted was used in conservative analyses.

[b] Conservative analysis 1 excluded SNPs associated with diabetes-related traits at $p<0.001$ (0.05/43).

[c] Conservative analysis 2 further excluded SNPs associated with any trait (except birthweight) at $p<5\times10^{-8}$.

[d] Conservative analysis 1 excluded SNPs associated with diabetes-related traits (any type of diabetes, 2 hour fasting glucose, HbA1c, insulin, and so on) at $p<6\times10^{-5}$ (0.05/820), or associated with any trait (except body size in adulthood) at $p<5\times10^{-8}$.

[e] Conservative analysis 2 further excluded SNPs associated with smoking, alcohol, or physical activity at $p<6\times10^{-5}$.

[f] Conservative analysis 3 further excluded SNPs associated with body size -related traits at birth at $p<6\times10^{-5}$.

[g] OR (95% CI) for one SD (0.5 kg) decrease in birthweight, or one SD (4.8 kg/m^2) increase in BMI in adulthood.

ESM Table 6. Risk of type 2 diabetes in relation to birthweight according to different methods

Methods	No. of SNPs	OR (95% CI)	*p* for risk estimate	*p* for heterogeneity
**IVW**	43	1.52 (1.13, 2.03)	0.005	<0.001
**Robust IVW**	43	1.60 (1.25, 2.04)	<0.001	<0.001
**Weighted Median**	43	1.51 (1.12, 2.05)	0.007	
**MR-Egger** [a]	43	1.67 (0.73, 3.83)	0.228	<0.001
**MR-PRESSO outlier-corrected** [b]	40	1.64 (1.32, 2.04)	<0.001	

IVW: inverse-variance weighted. MR-Egger: Egger regression of Mendelian randomization; MR-PRESSO: the Mendelian randomization pleiotropy residual sum and outlier approach.

[a] MR-Egger intercept: -0.002, *p* for directional pleiotropy: 0.811.

[b] MR-PRESSO detected rs40434, rs6925689, and rs13266210 as outliers. *p* for distortion of risk estimate by outliers was 0.533. Outliers were excluded from the outlier-corrected estimate.

ESM Table 7. Risk of LADA and type 2 diabetes in relation to adult BMI according to different methods using SNPs from UK Biobank

Methods	No. of SNPs	OR (95% CI)	*p* for risk estimate	*p* for heterogeneity
**LADA**				
IVW	734	1.45 (1.18, 1.79)	<0.001	<0.001
Robust IVW	734	1.41 (1.14, 1.74)	0.002	0.438
Weighted Median	734	1.51 (1.11, 2.04)	0.008	
MR-Egger [a]	734	2.69 (1.40, 5.18)	0.003	<0.001
MR-PRESSO outlier-corrected [b]	731	1.42 (1.18, 1.72)	<0.001	
**Type 2 diabetes**				
IVW	734	2.26 (2.06, 2.49)	<0.001	<0.001
Robust IVW	734	2.37 (2.19, 2.56)	<0.001	<0.001
Weighted Median	734	2.63 (2.37, 2.92)	<0.001	
MR-Egger [c]	734	3.32 (2.47, 4.47)	<0.001	<0.001
MR-PRESSO outlier-corrected [d]	725	2.33 (2.16, 2.51)	<0.001	

LADA: latent autoimmune diabetes in adults; IVW: inverse-variance weighted. MR-Egger: Egger regression of Mendelian randomization; MR-PRESSO: the Mendelian randomization pleiotropy residual sum and outlier approach.

[a] MR-Egger intercept: -0.011, *p* for directional pleiotropy: 0.05.

[b] MR-PRESSO detected rs10840606, rs2271189, and rs1046080 as outliers. Outliers were excluded from the outlier-corrected estimate (*p* for distortion of estimate by outliers: 0.816).

[c] MR-Egger intercept: -0.007, *p* for directional pleiotropy: 0.007.

[d] MR-PRESSO detected rs36090025, rs1002226, rs56094641, rs429358, rs10423928, rs61791109, rs329118, rs9366863, and rs849133 as outliers. *p* for distortion of risk estimate by outliers was 0.457. Outliers were excluded from the outlier-corrected estimate.

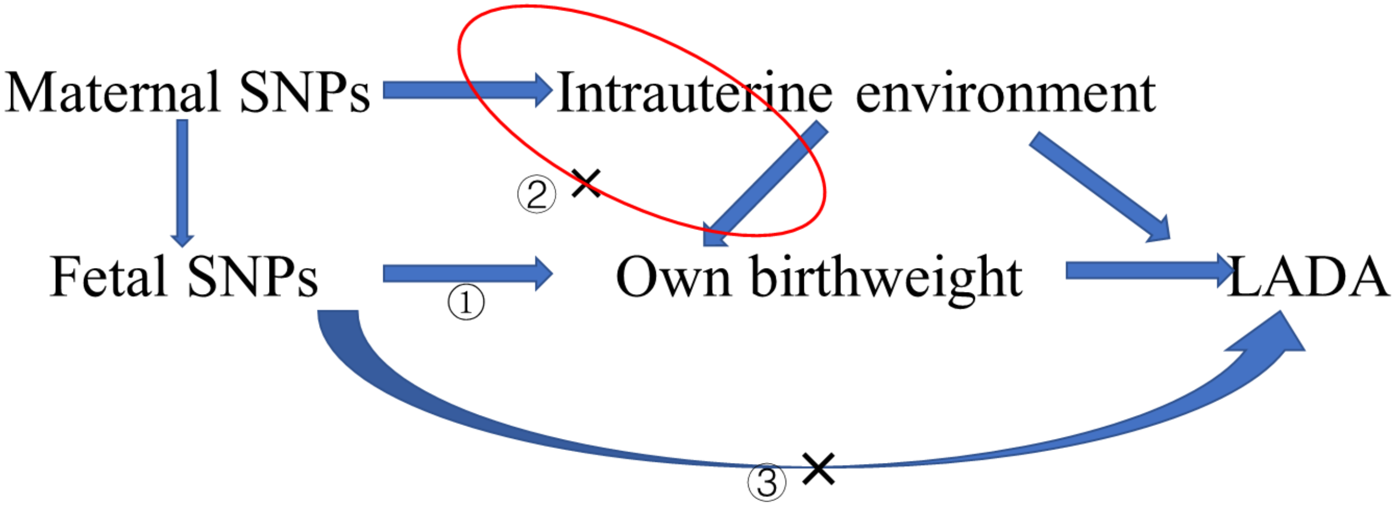

ESM Fig. 1 Reasons for the selection of SNPs for birthweight in the main analysis

LADA: latent autoimmune diabetes in adults.

SNPs used as instrumental variables for own birthweight should be associated with own birthweight (pathway ①), should not be associated with confounders (intrauterine environment, pathway ②) on the own birthweight-LADA association, and should not affect LADA directly (pathway ③).

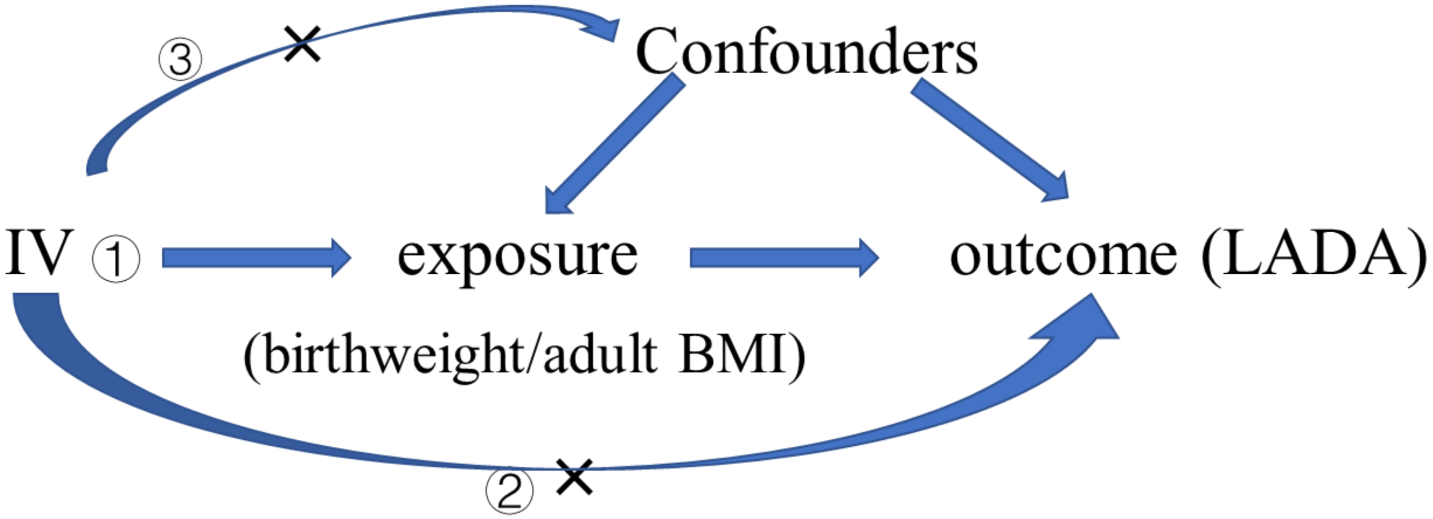

ESM Fig. 2 IV assumptions

IV: instrumental variable; LADA: latent autoimmune diabetes in adults.

IV can only affect the outcome through the exposure (①), not through a direct pathway (②) to the outcome or via a confounder (③).

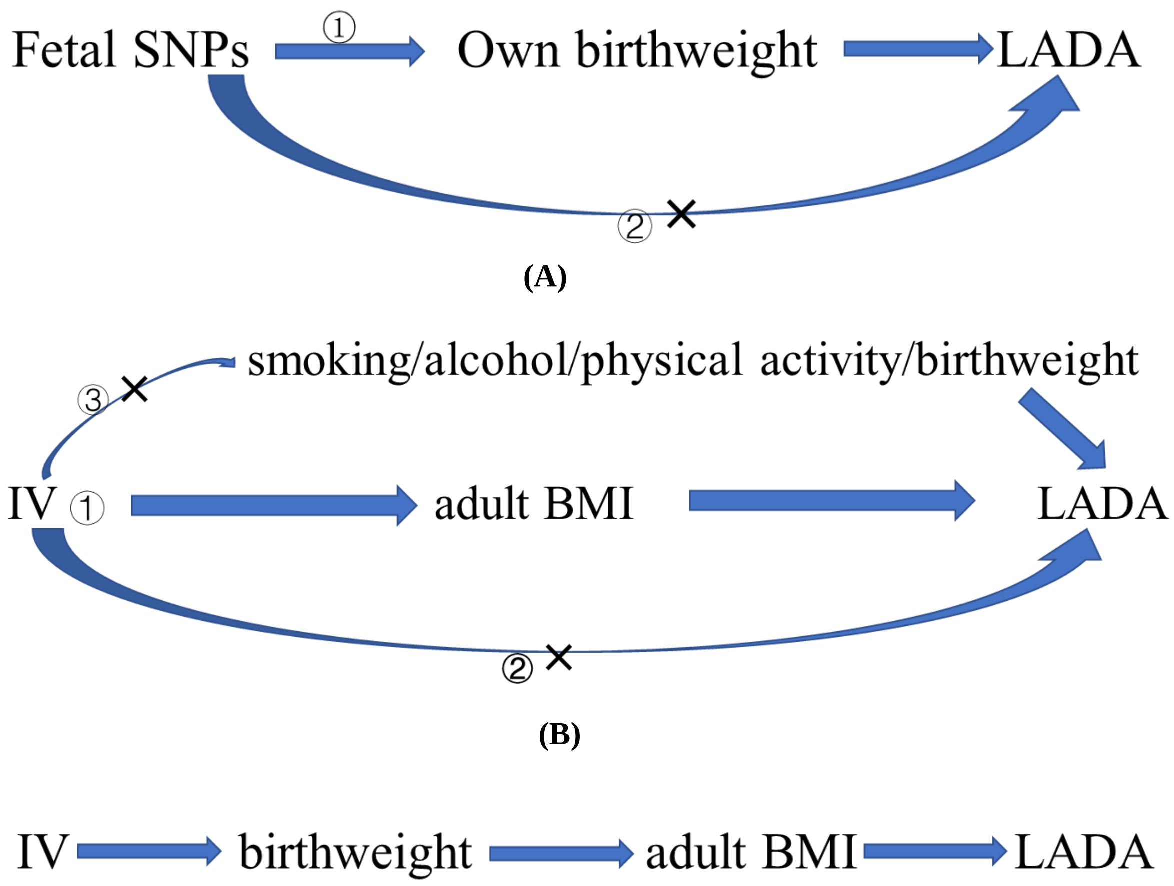

(C)

ESM Fig. 3 Directed acyclic graphs showing reasons for conservative analyses

IV: instrumental variable; LADA: latent autoimmune diabetes in adults.

(A) The IV for birthweight should not affect LADA directly (pathway ②). (B) The IV for adult BMI should not affect LADA directly (pathway ②) or through other pathways (pathway ③). (C) The IV might affect birthweight and adult BMI through the same pathway and there is no violation of IV assumption in this scenario.

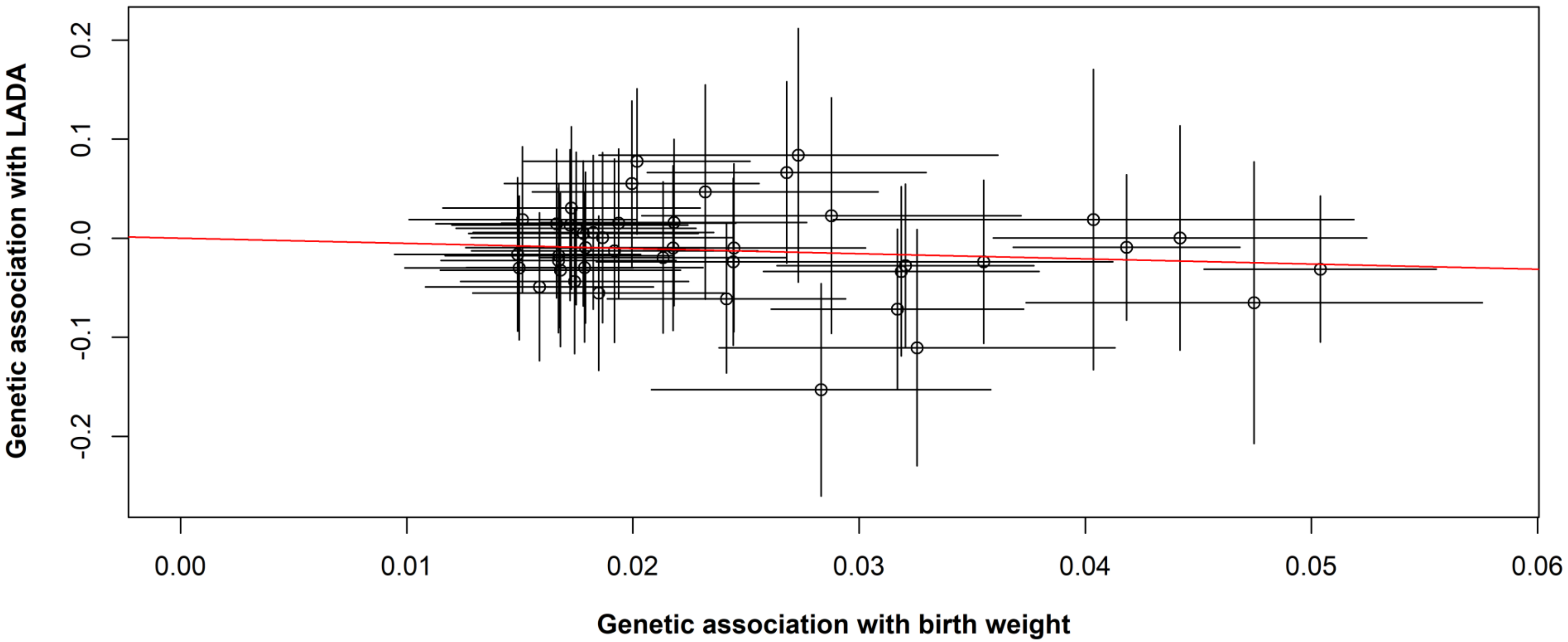

ESM Fig. 4 Scatter plot for associations of 43 SNPs with birthweight and LADA.

LADA: latent autoimmune diabetes in adults. The slope of the red line is the log OR of increase in LADA risk one SD (0.5 kg) increase in birthweight, based on the inverse-variance weighted method.

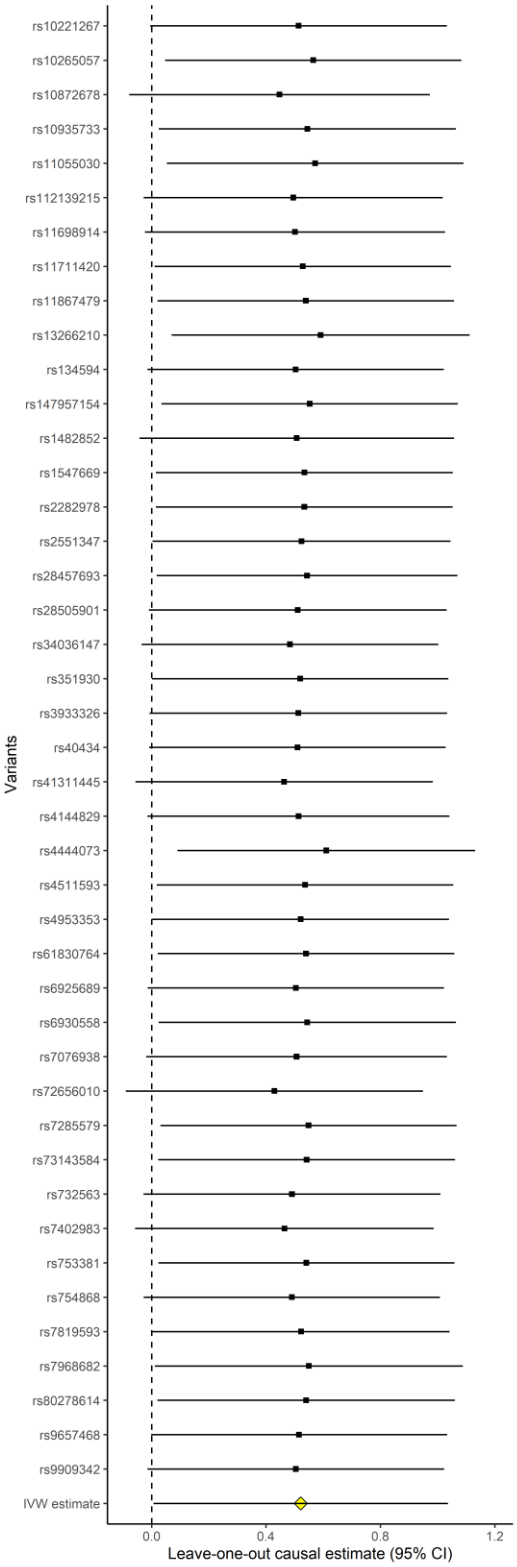

ESM Fig. 5 Leave-one-out analysis for risk of LADA one SD decrease in genetically determined birthweight

LADA: latent autoimmune diabetes in adults.

Each SNP included in the main analysis was left out in turn, leaving 42 SNPs in each causal estimate. Causal estimate was presented as log ORs and corresponding 95% CIs.

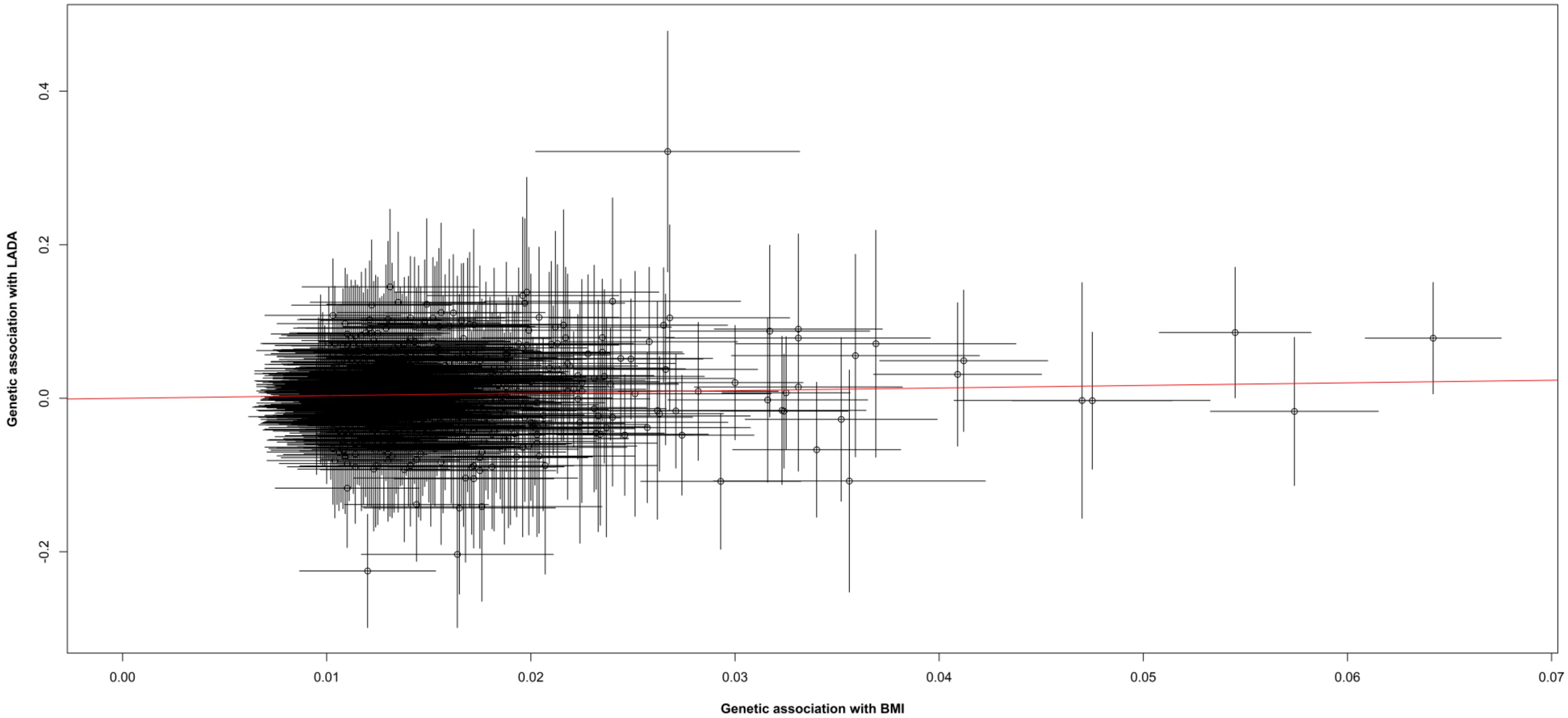

ESM Fig. 6 Scatter plot for associations of 820 SNPs with BMI and LADA

LADA: latent autoimmune diabetes in adults.

The slope of the red line is the log OR of increase in LADA risk one SD (4.8 kg/m^2) increase in BMI, based on the inverse-variance weighted method.

Methods	No. of SNPs	OR (95% CI)	*p* for risk estimate	*p* for heterogeneity
IVW	818	1.44 (1.19, 1.75)	<0.001	0.071
Robust IVW	818	1.42 (1.15, 1.76)	0.001	0.705
Weighted median	818	1.40 (1.02, 1.91)	0.034	
MR-Egger	818	2.18 (1.21, 3.94)	0.010	0.075
MR-PRESSO raw estimate	818	1.44 (1.19, 1.76)	<0.001	

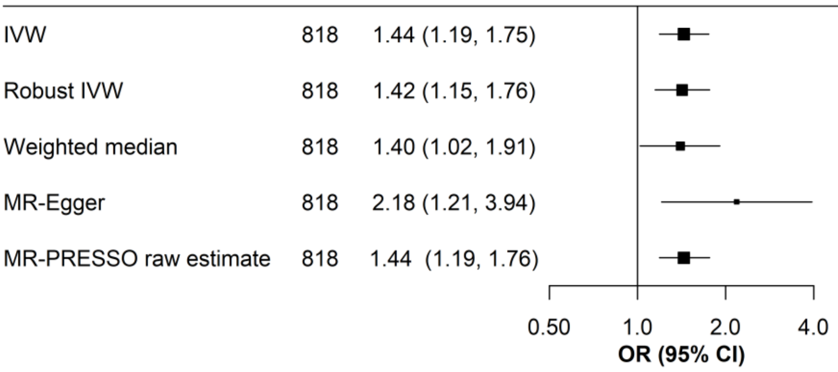

ESM Fig. 7 The risk of LADA in relation to one SD (0.5 kg) increase in adult BMI based on 818 SNPs

IVW: inverse-variance weighted. MR-Egger: Egger regression of Mendelian randomization; MR-PRESSO: the Mendelian randomization pleiotropy residual sum and outlier approach; LADA: latent autoimmune diabetes in adults.

MR-Egger intercept: -0.007, *p* for directional pleiotropy: 0.149.

MR-PRESSO detected no outlier and the raw estimate was reported.

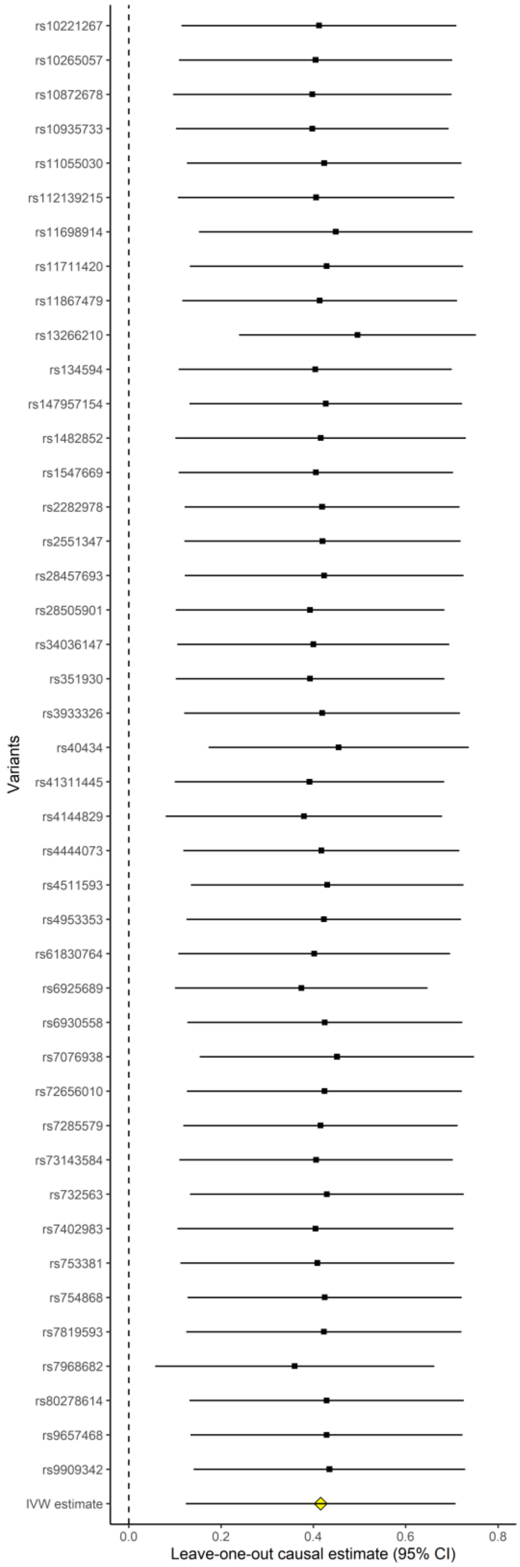

ESM Fig. 8 Leave-one-out analysis for risk of type 2 diabetes one SD decrease in genetically determined birthweight

Each SNP included in the main analysis was left out in turn, leaving 42 SNPs in each causal estimate. Causal estimate was presented as log ORs and corresponding 95% CIs.

References

[1] Warrington NM, Beaumont RN, Horikoshi M, et al. (2019) Maternal and fetal genetic effects on birth weight and their relevance to cardio-metabolic risk factors. Nat Genet 51(5): 804-814. 10.1038/s41588-019-0403-1

[2] Juliusdottir T, Steinthorsdottir V, Stefansdottir L, et al. (2021) Distinction between the effects of parental and fetal genomes on fetal growth. Nat Genet 53(8): 1135-1142. 10.1038/s41588-021-00896-x

[3] Yengo L, Sidorenko J, Kemper KE, et al. (2018) Meta-analysis of genome-wide association studies for height and body mass index in approximately 700000 individuals of European ancestry. Hum Mol Genet 27(20): 3641-3649. 10.1093/hmg/ddy271

[4] Kamat MA, Blackshaw JA, Young R, et al. (2019) PhenoScanner V2: an expanded tool for searching human genotype-phenotype associations. Bioinformatics 35(22): 4851-4853. 10.1093/bioinformatics/btz469

[5] Staley JR, Blackshaw J, Kamat MA, et al. (2016) PhenoScanner: a database of human genotype-phenotype associations. Bioinformatics 32(20): 3207-3209. 10.1093/bioinformatics/btw373
